# Causal involvement of the left angular gyrus in higher functions as revealed by transcranial magnetic stimulation: a systematic review

**DOI:** 10.1007/s00429-022-02576-w

**Published:** 2022-10-19

**Authors:** Jennifer Wagner, Elena Rusconi

**Affiliations:** Department of Psychology and Cognitive Sciences, University of Trento, Corso Bettini, 31, 38068 Rovereto, TN Italy

**Keywords:** Angular gyrus, Transcranial magnetic stimulation, Theta burst stimulation, Cognition, Left hemisphere

## Abstract

Transcranial magnetic stimulation (TMS) is a non-invasive technique that can transiently interfere with local cortical functioning, thus enabling inferences of causal left AG involvement in higher functions from experimentation with healthy participants. Here, we examine 35 studies that measure behavioural outcomes soon after or during targeting TMS to the left AG, by design and as documented by individual magnetic resonance images, in healthy adult participants. The reviewed evidence suggests a specific causal involvement of the left AG in a wide range of tasks involving language, memory, number processing, visuospatial attention, body awareness and motor planning functions. These core findings are particularly valuable to inform theoretical models of the left AG role(s) in higher functions, due to the anatomical specificity afforded by the selected studies and the complementarity of TMS to different methods of investigation. In particular, the variety of the operations within and between functions in which the left AG appears to be causally involved poses a formidable challenge to any attempts to identify a single computational process subserved by the left AG (as opposed to just outlining a broad type of functional contribution) that could apply across thematic areas. We conclude by highlighting directions for improvement in future experimentation with TMS, in order to strengthen the available evidence, while taking into account the anatomical heterogeneity of this brain region.

**Supplementary Information:**

The online version contains supplementary material available at 10.1007/s00429-022-02576-w.

## Introduction

This systematic review provides a synthesis of evidence from transcranial magnetic stimulation (TMS) studies including an active stimulation condition of the angular gyrus (AG) in the left hemisphere and related measures of behavioural outcomes. TMS is a noninvasive stimulation technique which can interfere with a limited area of cortical tissue under a stimulating coil positioned on the scalp (Walsh and Pascual-Leone 2003). The literature offers a wealth of studies that could, by design or incidentally, have interfered with left AG functioning. However, the main objective of this review is to enucleate and synthesize an inner core of evidence, by considering studies where the left AG is explicitly targeted and unequivocally localized on individual magnetic resonance images (MRI) before applying stimulation. The outcomes of this selective synthesis could then be easily complemented and contrasted with less specifically targeted or localized, but still potentially relevant literature, in future endeavours.

In this section, we will first introduce a few basic anatomical concepts pertaining to the AG. This will be followed by a brief overview of functional domains that have been linked to the AG from various sources of evidence. Finally, we will provide the rationale of our review.

### Anatomical characterization of the AG

**Fig. 1:**
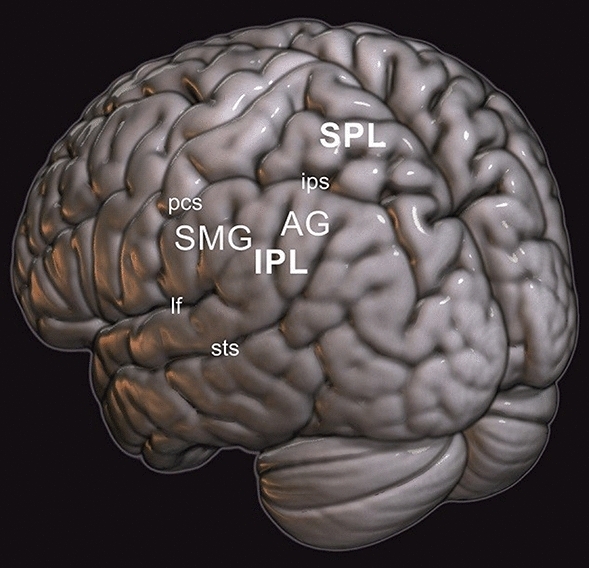
Localization of AG in the posterior part of the IPL and macro-anatomical landmarks. Sulci and fissures are indicated in lowercase. *lf* lateral fissure, *pcs* post-central sulcus, *ips* intraparietal sulcus

The AG is a part of the parietal cortex, which is divided in two main regions: the anterior parietal cortex (APC) and the posterior parietal cortex (PPC). The PPC can be further divided in three broad regions, with distinctive cyto-, myelo-, recepto-architectonic and connectivity profiles: the superior parietal lobule (SPL), the intraparietal sulcus (IPS) and the inferior parietal lobule (IPL). Within the IPL, two subdivisions can be identified based on macro-anatomical landmarks: the supramarginal gyrus (SMG) rostrally and the AG caudally (see Fig. 1). In a lateral view of the brain, the SMG arches over the upturned end of the lateral fissure and is delimited anteriorly by the post-central sulcus, which separates it from the APC, and dorsally by the IPS. The AG arches over the posterior end of the superior temporal sulcus (STS), which takes the name of angular sulcus. The AG is delimited anteriorly by the SMG and the primary intermediate sulcus of Jessen (when present), dorsally by the IPS and, posteriorly, it transitions into the occipital cortex without a definite landmark (Grey 1901; Ono et al. 1990; Caspers and Zilles 2018). Essentially, these two regions correspond to Brodmann’s (1909) area (BA) 40 and 39, and to von Economo and Koskinas’ (1925) area PF and PG, respectively. Based on recent quantitative analyses of local cytoarchitecture, the two IPL regions may, in fact, be composed of a mosaic of smaller and heterogeneous areas, two of which are located in the caudal portion of the IPL, in correspondence with the AG (PGa and PGp; Caspers et al. 2006, 2008). The patterns of long-range functional and structural connectivity characterizing spatially adjacent but cytoarchitectonically distinct regions of the AG differ not only from those of the banks of the IPS, which borders the IPL dorsally, but also between themselves. For example, the rostral part of the AG is both functionally and structurally connected with ventral premotor areas, ventrolateral prefrontal cortex and basal ganglia, whereas the caudal part of the AG is more strongly linked with the ventromedial prefrontal cortex, the posterior cingulate and the hippocampus (Uddin et al. 2010). In addition, a portion of the fiber bundles providing long-range connections from the IPL show different lateralization (Caspers and Zilles 2018; Seghier 2013). For example, whereas part of the superior longitudinal fascicle (SLF; in particular, SLF III), connecting the IPL with ventrolateral premotor and prefrontal cortices and posterior temporal areas, is right lateralized (Thiebaut de Schotten et al. 2011), parts of the middle longitudinal fascicle, connecting IPL regions with the temporal lobe up to the temporal pole, are left lateralized (in particular those originating in the AG; Makris et al. 2013). In other words, current knowledge of macro- and micro-anatomy of the AG points in the direction of both an internal differentiation of functional roles and hemispheric specialization.

### Functional characterization of the AG

A picture of functional heterogeneity emerges also from anatomically-focused reviews (e.g. Cabeza et al. 2012; Seghier 2013; Humphreys et al. 2021). For example, in a broad analysis focused on the AG, Seghier (2013) identifies seven thematic clusters emerging from functional imaging studies with healthy human participants. The clusters correspond to distinct functional domains or cognitive states and operations and have been consistently linked to changes in AG activity. Of these, semantic processing, reading and comprehension are associated with recruitment of the left AG in particular, regardless of the modality with which the stimuli are presented, and especially when concept retrieval and integration are required. Additionally, both the left and the right AG have been strongly implicated in the default mode network, number processing, attention and spatial cognition, memory retrieval, and social cognition. Overall, the available evidence suggests a possible role of the AG in revisiting and manipulating conceptual knowledge when the mind is not engaged in exogenous tasks, and involvement in verbal working memory, autobiographic and episodic memory, as well as levels of subjective confidence in retrieval accuracy. The left and right AG appear to be strongly involved in both verbal and non-verbal theory-of-mind tasks, often together with contiguous posterior temporal areas, with a list of potential functional roles—some specific to social cognition, others related to memory and contextual knowledge. Distinct roles have been proposed for the left and the right AG in number processing, such as supporting the retrieval of arithmetic facts (especially those learnt in a verbal format) and providing visuospatial support to calculation, respectively. A similar differentiation has been found in attention and spatial cognition, where the right AG would be especially critical for re-orienting and maintaining attention and the left AG for integrating spatial information with language and conceptual knowledge. In tasks requiring conflict resolution, there is robust evidence for a right AG involvement whereas left AG appears to be recruited only in the presence of a strong contextual/semantic conflict. In a more recent review, Humphreys et al. (2021) propose a unifying account of (mainly left) AG contributions to episodic and semantic memory as a case study for the general idea that an associative brain region could perform basic neurocognitive computations to serve different functional domains rather than being a mosaic of tightly packed neighbouring subregions with different domain-specific functions (e.g. Cabeza et al. 2012; Humphreys and Lambon Ralph 2017). More specifically, they suggest that the left AG may work as an online dynamic buffer of multisensory representations with spatiotemporal extension. It would not act as a storage of long-term (semantic or episodic) memories, but rather as a binding point for current information from different sensory modalities and/or spatiotemporal frameworks. This would allow, among other things, the formation of an explicit and up-to-date representation of the internal and external world that may be necessary to keep track of time-extended events or activities and relate them to one’s own internal state and pre-existing memories. Left AG computations could thus provide a fundamental contribution to our adaptive behaviour and ability to relate to and learn from current experiences. Humphreys et al. (2021)’s proposal raises the question of whether and how such integrative buffering function may play a role in tasks that tap on other cognitive domains related to left AG integrity.

Consistent with the neuroimaging literature, in neurological patient studies left AG lesions have been implicated in deficits involving several higher functions for a long time (e.g. Dejerine, 1891; Liepmann 1920; Gerstmann 1940). Also, the quest for a possible integrative, single-process explanation of the left AG contribution is still on-going. For example, in the “angular gyrus syndrome” (Triarhou 2008), also known as Gerstmann syndrome and first described in a patient in 1924 (Gerstmann, 1924; English translation available in Rusconi and Cubelli 2019), a tetrad of symptoms has been described, potentially involving four different cognitive domains: acalculia, agraphia, finger agnosia and left–right disorientation. Based on post-mortem examinations, this cluster of symptoms was originally linked to a single lesion located in the area of transition between the dominant AG and the second occipital convolution (Gerstmann 1940), although later analyses have suggested either a more rostral and dorsal positioning of the key lesion within the AG (Strub and Geschwind 1983; Mazzoni et al. 1990), or a subangular placement (Mayer et al. 1999). Soon after its first description, a quest for an underlying basic functional deficit that could explain the heterogeneous constellation of symptoms started, with several proposals put on the table throughout the years (e.g. Conrad 1932; Gerstmann 1940; Gold et al. 1995; Mayer et al. 1999; Ardila 2014). What most of the proposals have in common is a special connection with one functional domain (e.g. involving body awareness or space processing) which, when compromised, would then exert primacy over aspects of the other affected domains and prevent their normal functioning. Alternative proposals have ascribed the co-occurrence of deficits to anatomical proximity of functionally distinct regions within the left AG rather than to a shared anatomo-functional substrate or common operation between domains (e.g. Benton 1992; Wingard et al. 2002) and/or to disconnection after lesion to a subcortical convergence hotspot for intraparietal connecting fibers from separate subregions of the left PPC (e.g. Rusconi et al. 2009; Kleinschmidt and Rusconi 2011). Studies with direct electrical stimulation during awake open-brain surgery have provided relevant converging evidence. For example, Morris et al. (1984) reproduced impairments similar to those reported in Gerstmann syndrome without any associated extraneous impairments, by stimulating contiguous sites (but not a single site) located in the left AG of an epileptic patient. Stimulation sites outside the left AG producing related impairments produced also associated extraneous impairments (see also Roux et al. 2003, for similar evidence in a group of patients with brain tumour; and Vaddiparti et al. 2021, for the compatible finding of a hotspot of sites causing Gerstmann syndrome-like impairments in the left inferior SPL, at the border with the left AG, in a patient with a primary epileptic focus centered in the IPL). Overall, the evidence from direct electrical stimulation studies appears consistent with a causal and specific involvement of sites in the left AG in Gerstmann syndrome but speaks against models proposing a shared cortical substrate with a common basic functional impairment. Conversely, it is compatible with the proximity and the bottleneck disconnection models for the co-occurrence of deficits in separate domains after left AG lesion. Last but not least, the involvement of portions of the left AG in other domains has been reported in the same and other studies using direct electrical stimulation (e.g. Morris et al. 1984; Roux et al. 2003; Desmurget et al. 2009). Direct electrical stimulation typically enables a finer-grained appreciation of the neural substrate supporting cognitive functions within a region than lesion studies and shares with lesion studies the advantage of enabling causal inferences between brain and behaviour. Unlike lesion studies, the local alteration of brain activity is transient and unlikely to produce functional reorganization. However, it also shares with lesion studies two important limitations: local alteration of brain function may exert behaviourally relevant effects on distant but connected regions (i.e. behavioural effects may be related only indirectly to local alterations) and it can only be applied to neurological patients (i.e. people whose brains have medically relevant alterations). Additionally, it requires invasive procedures.

### Review rationale

Noninvasive experimental counterparts of the lesion and direct electrical stimulation approaches are also available, enabling a transient and targeted perturbation of brain functioning in healthy participants, who can therefore act as controls for their own experimental performance. Within the family of noninvasive stimulation techniques, which also include transcranial electrical stimulation (tES) techniques, TMS affords greater temporal and spatial focality. It is therefore the current technique of choice to localize brain function, whereas typical tES studies may not be as informative in this respect (see Karabanov et al. 2019). A standard 70-mm butterfly coil can stimulate about 1–2 cm^2^ of cortex under its central junction, with the effects of a single pulse of TMS lasting only up to a few milliseconds, and the behavioural resolution of TMS effects can range from 0.5 cm^2^ to 1.5 cm^2^ apart (Walsh and Pascual-Leone 2003; O’Shea and Walsh 2007). Spatial, temporal and behavioural resolution, however, are also crucially dependent on the stimulated tissue, stimulation protocols and parameters (see Sandrini et al. 2011 for a detailed overview). Evidence from TMS studies can be informative about a possible AG involvement during cognitive or behavioural tasks thanks to the superficial location of this region in the brain. Indeed, TMS performed with typical coils can directly stimulate cortical tissue up to 2–3 cm from the skull surface. Significant behavioural effects can be taken to indicate a local involvement in crucial neural computations as required by the task at hand, although involvement by virtue of TMS effects on functionally connected circuits is also possible (Driver et al. 2009). In this review, we will include studies employing TMS both in an online mode, where it is used to interfere with activity in the target region during task performance, and in an off-line mode, where it is used to induce changes in local cortical excitability that can outlive the stimulation window. Of great importance is the localization method used to identify the coil placement site on a participant’s scalp, as it determines the likelihood of consistently delivering stimulation on the target anatomical region and consequently the power of a study (e.g. Sack et al. 2009; Ahdab et al. 2010). In rare cases, functional MRI (fMRI) activation data are available for each participant in the same task that will be probed with TMS, or in a localizer task, thus allowing interindividual differences in functional organization within the region of interest to be considered. For the purpose of this review, in such cases the macro-anatomical region of interest should be limited a priori to the left AG (rather than broadly to IPL or PPC) before selecting individual stimulation sites (e.g. on the basis of functional connectivity with a hippocampal seed region, as in Thakral et al. 2020 and Hermiller et al. 2019). More often, however, the neural hotspot is identified on individual brains with reference to well-known macro-anatomical landmarks (e.g. the gyrus running around the posterior boundary of the STS, lateral to the posterior IPS; as in Rusconi et al. 2005) and/or coordinates based on previous fMRI findings (e.g. average peak coordinates in stereotaxic space from single studies or meta-analyses, which will then be back-transformed to the individual MRI native space; as in Varnava et al. 2013). Because these methods can control a priori for anatomical interindividual variability, they will be included in our review. In some cases, however, MRI images are not available for some or all participants, and localization of the scalp stimulation site may be performed with reference to the 10–20 EEG system (e.g. typically P3 for the left PPC) and/or to a standard brain image (e.g. Rusconi et al. 2007), or via scalp coordinates (e.g. locating first a starting point on the scalp of the participants such as the inion and moving by a fixed distance based on pilot testing or previous studies, e.g. 9 cm dorsal 6 cm lateral; Walsh et al. 1999). In all these cases, neither interindividual differences in anatomical organization nor interindividual differences in functional organization can be controlled for a priori. Therefore, studies employing such localization methods will not be included. The use of a TMS functional localizer in the absence of an a priori individual MRI image represents a borderline case for a review such as this one, as it may control for interindividual variability in functional localization but not for anatomical variability. Indeed, studies using the same functional localizer procedure have reported targeting different parietal regions (e.g. Ashbridge et al. 1997, Fig. 7; and Walsh et al., 1999, locate their stimulation site in the right and left SPL/IPS; Rushworth et al. 2001, locate their stimulation site in the right and left AG). We have therefore opted for including studies with a TMS functional localizer on condition that the stimulation site was also anatomically localized on MRI images for all participants. We have additionally opted to limit our search to studies with an explicit mention of the AG (or BA39 or PGa or PGp) in their abstract, title or associated keywords, as this approach enables focusing on the most anatomically specific evidence. It has the disadvantage of missing potentially relevant evidence that is not framed within mainstream AG research or that is routinely associated to other labels. These may be superordinate to the AG, and therefore point to a larger region (e.g. PPC or IPL), or may be more imprecise (e.g. TPJ, often used in the literature to indicate a patch of cortex including a limited portion of the AG, as well as neighbouring portions of SMG and STS; it can also be used to indicate an area of the SMG around the end of the Sylvian fissure; e.g. Cabeza et al. 2012; Donaldson et al. 2015). In some of these cases, a check of the average stereotaxic coordinates of the stimulation site, when provided, may reveal that the site is likely to fall within the boundaries of the AG, according to atlases in use. However, average coordinates, in the absence of specific labelling or explicit anatomical criteria, may be insufficient to conclude that AG stimulation was carried out consistently across participants.

Against a backdrop of known anatomical heterogeneity on the one hand and the constant strive to identify a unique function or computational contribution for the left AG on the other hand, a thorough and systematic examination of the literature on the causal involvement of the left AG in healthy brains appears to be a crucial stepping stone. Such evidence, collected across domains and identified solely on the basis of its anatomical relevance (i.e. regardless of consistency with one or other theoretical proposal and of its thematic focus), should receive particular attention and be accounted for by any theoretical proposals seeking an all-encompassing and sufficiently detailed explanation of the left AG role in cognition. Functional heterogeneity, with the characteristics confirmed and/or specified by causal evidence, can either pose a major challenge for an all-encompassing theoretical approach or provide cogent clues for theorization efforts, which so far have been initiated from within specific domains and/or have been built on non-systematic selections of evidence.

## Methods

We conducted a systematic review of the literature that investigated cognitive or behavioural effects of TMS stimulation over the left AG. This systematic review was conducted following PRISMA guidelines where suitable (Page et al. 2021). The resources created for and obtained from this study are available on the Open Science Framework at the following link: https://osf.io/8f2m3/.

### Data sources

A first search in PubMed, Scopus and Web of Science was conducted on 10th February 2022 with the terms indicated below in italics; it was subsequently extended and updated on 30th April 2022, with the following strings and filters:

#### PubMed:

Search: (*angular gyrus[Title/Abstract] OR BA39[Title/Abstract]* OR PGa[Title/Abstract] OR PGp[Title/Abstract]*) AND (transcranial magnetic stimulation[Title/Abstract] OR TMS[Title/Abstract]* OR theta burst stimulation[Title/Abstract] OR TBS[Title/Abstract]*).*

Language = *English*; Exactkeyword = *Human*; *Humans*.

#### Scopus:

Search: *TITLE-ABS-KEY ((angular AND gyrus OR BA39* OR PGa OR PGp*) AND (transcranial AND magnetic AND stimulation OR tms* OR theta AND burst AND stimulation OR tbs*)) AND (LIMIT-TO(EXACTKEYWORD, “Human”) OR (LIMIT-TO(EXACTKEYWORD, “Humans”)) AND (LIMIT-TO(LANGUAGE, “English”)).*

#### Web of Science:

Topic Search (i.e. in Title, Keyword, Abstract, KeywordPlus): *TS* = *((angular gyrus OR BA39* OR PGa OR PGp*) AND (transcranial magnetic stimulation OR tms* OR theta burst stimulation OR tbs*)).*

Language = *English*.

The final search returned a total of 405 hits (88 in PubMed, 164 in Scopus and 153 in Web of Science). There were no internal duplicates within each database and 172 external duplicates between the databases. After removing duplicates from these lists, a total of 233 results underwent screening.

### Data screening and eligibility

**Fig. 2:**
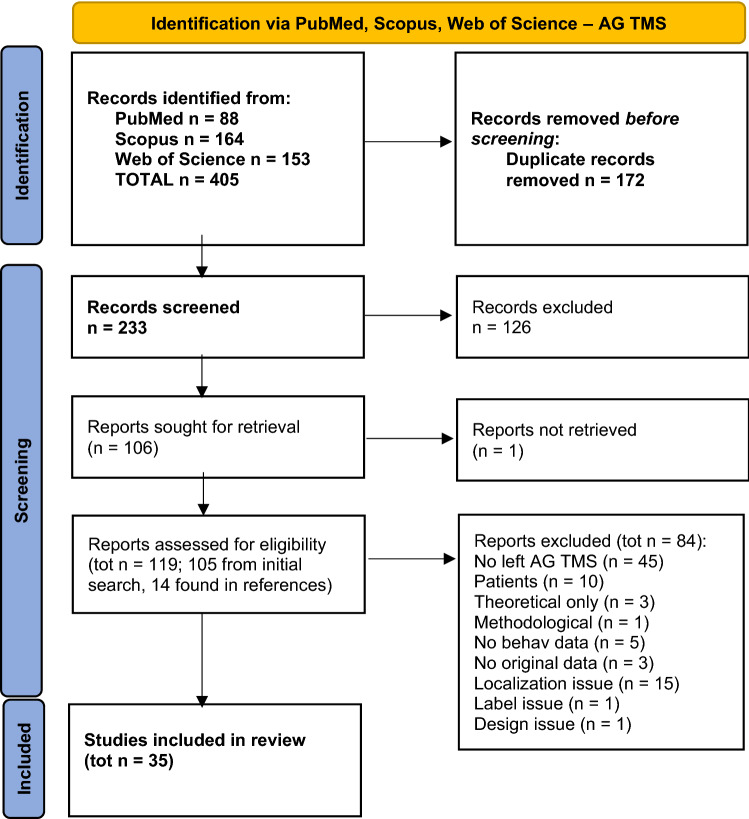
PRISMA 2020 flow diagram for new systematic reviews

Figure 2 summarizes the screening and selection workflow. During the screening, criteria were applied leading to the exclusion of papers with no original data (e.g. editorials, reviews, reanalyses, corrigenda) or no healthy adult participants, methodological papers and papers without behavioural measures, studies where no TMS stimulation on the left AG was administered (either as a main focus of the study or as an active control site), where AG localization could not be performed on individual MRIs for all participants, or where the provided information on and labelling of the stimulated site suggested possible inconsistencies across participants, and/or a broad focus on IPL/PPC (e.g. when average coordinates were provided–with or without measure of dispersion—compatible with AG stimulation but the site was broadly labelled as IPL or TPJ, and/or when individual coordinates suggested that different regions had been stimulated). All original studies with healthy adult participants not meeting the exclusion criteria, with at least one condition where off-line or online TMS was delivered to the left AG, and measures to assess the presence of left AG stimulation behavioural–cognitive effects, were included in the review. Both studies reporting significant effects and studies reporting null effects of AG stimulation were included. After screening of the abstracts, a list of 106 research papers was retained for full-text screening. One of the papers could not be retrieved and 14 extra papers were found in references, bringing the total to 119. Full-text screening identified 84 studies (of which 12 found in references) that fulfilled the exclusion criteria outlined above (reasons for exclusion and number of papers are detailed in Fig. 2) and a total of 35 studies that fulfilled the inclusion criteria. Therefore, 35 studies were included in the review.

### Data extraction

The following information was collected from the records: year of publication, sample characteristics (age, gender, handedness) and size (recruited, excluded, analysed), stimulation protocol (e.g. single/repeat session, stimulation frequency, train duration, stimulation intensity, coil angle, type and size, localization method, site coordinates, control site), cognitive–behavioural testing protocol characteristics (e.g. main task, control task, linked function, dependent variable, effect measure) and analyses and outcomes (statistical methods, significance, effect size, conclusions drawn by the authors). For the labelling of functions, we used terms that are widely accepted in the literature, such as language, memory, visuospatial attention, motor planning, numerical cognition and bodily awareness. The list of studies included in this review and a selection of the extracted data is provided in Supplementary Table 1.

### Study quality

The QUADAS quality assessment tool (Whiting et al. 2003; 2006) was adapted for this review to document the steps taken by each paper to avoid bias and justify and validate the protocols (see also Forkel et al. 2022). The following criteria were used in a rating of publications for internal use at the general evaluation stage: (1) sufficient detail provided to reproduce the protocol (e.g. 1 = some details missing, 2 = sufficient details provided, 3 = materials publicly available), (2) clearly defined localization and stimulation method, (3) reporting of the sample characteristics, including handedness and (4) robustness of the statistical approach. Due to the varied nature of research goals and protocols, no papers were excluded on the basis of these criteria.

## Results

In this section, we will provide a general picture emerging from the included studies. Given the heterogeneity in approaches and reporting standards it is not possible to provide exact information for variables such as age (e.g. some studies report indices of central tendency and dispersion, others only central tendency, others only range, others omit age information) or number of independent experiments, stimulation sessions or unique participants (e.g. a few studies adopted a mixed between-/within-participants approach, with some participants assigned to more than one experimental condition, or some participants taking part in more than one experiment reported separately in the study with others participating only to one experiment), but we will nonetheless give a gist of some of the most relevant characteristics in this section. For a detailed synthesis by study and thematic area, we refer the reader to Supplementary Table 1.

### Participants

Based on the information provided in the included papers, most of the participants were young adults (18–35 years) and very few middle aged, with an overall even proportion of males and females. Reported sample sizes vary between 3 and 69 per experiment, with about 55% of them positioned between 10 and 20; larger sample sizes are typical of studies with a between-participants design.

### Localization methods

The selected studies applied at least one of these methods, to locate the left AG on each included participant: (1) fMRI-guided neuronavigation based on individual-specific coordinates constrained a priori to the left AG (e.g. Thakral et al. 2020), (2) individual MRI-guided neuronavigation based on macro-anatomical landmarks (a priori, or post hoc, if combined with an a priori functional localizer; e.g. Reader et al. 2018; Rushworth et al. 2001), 3) MRI-guided neuronavigation based on average coordinates in stereotaxic space (from meta-analyses or single studies), projected on the normalized brain of a participant and then back-transformed to coordinates in native space for site identification (e.g. Varnava et al. 2013).

Most frequently, in these studies the left AG target was identified a priori on a participant’s MRI based on coordinates in Talairach/MNI space from meta-analyses or single studies related to Broadmann’s areas (BA39), or via macro-anatomical landmarks. The stimulated region is generally referred to as AG or BA39 (though in a few cases qualifiers such as middle AG or dorsal AG, are used to indicate AG subregions; e.g. Sliwinska et al. 2015).

### Thematic areas

Six thematic (cognitive) domains were identified, each with an unequal number of studies. The most frequently investigated functions were memory (*n* = 10) and language (*n* = 8), followed by number processing (*n* = 7) and visuospatial attention (*n* = 6), and finally by motor planning (*n* = 2) and body awareness (*n* = 2). Rusconi et al. (2005) contained two experiments tackling separate domains (body awareness and number processing), none of which was devised as a control task for the other. It was included only once in the count, as a paper on body awareness, but it will be in part discussed also under number processing.

### Stimulation site by thematic area

**Fig. 3:**
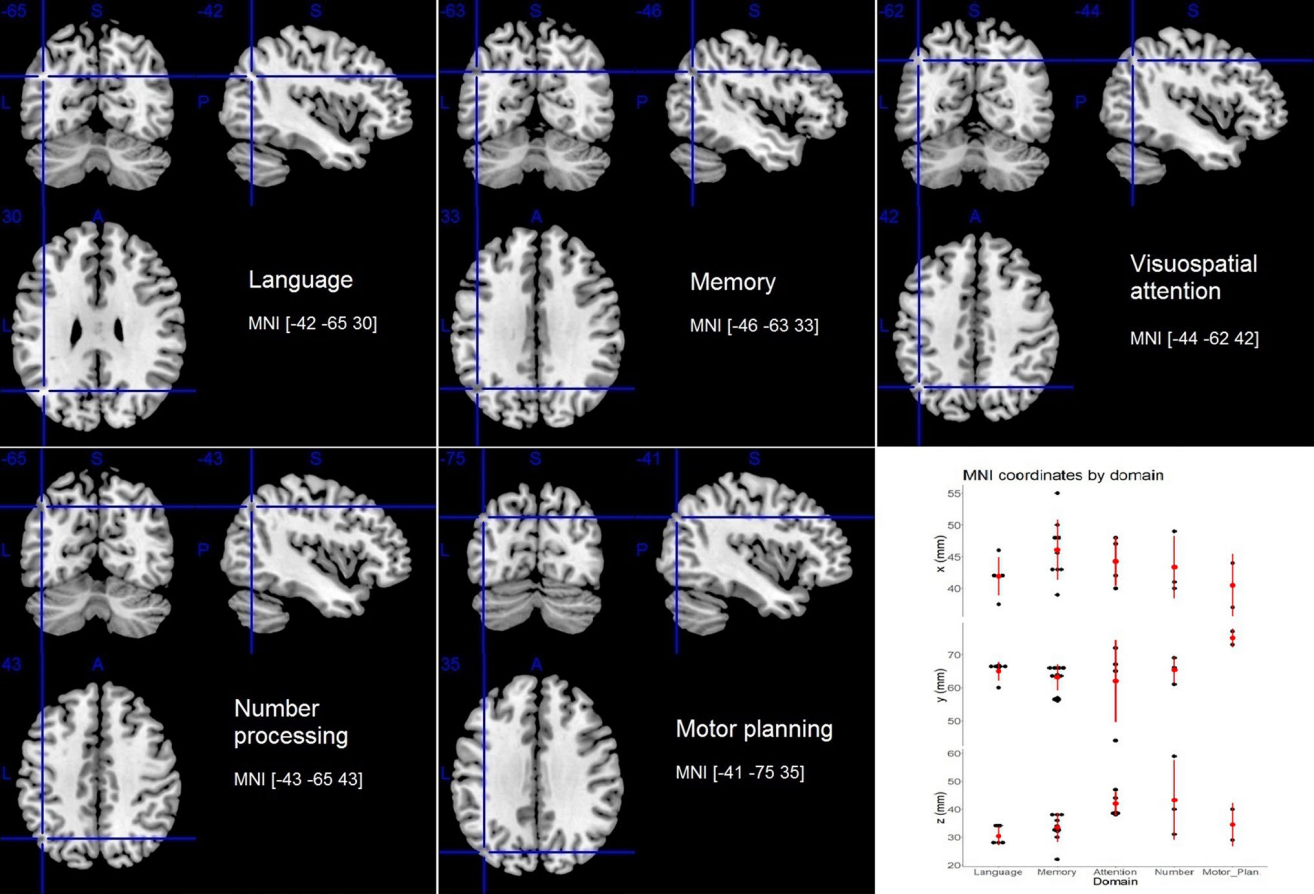
The figure shows, on a brain template (Rorden and Brett 2000), the mean MNI coordinates of the targeted site by domain (where available, see Supplementary Table 2); in the lower right panel, the mean x, y, and z coordinates (red dots) are plotted by domain along with their standard deviation (vertical red lines indicate ± 1 standard deviation from the mean) and with the original values (black dots) of the MNI coordinates for each study contributing to the mean

Due to the size and heterogeneity of the left AG and the potential resolution (both spatial and functional) of TMS, it is possible that research efforts in different thematic areas have tended to focus on different parts of the same macro-anatomical region. Traditional labelling practices do not enable such a check (see “Localization methods”), and more recent labelling proposals (e.g. using PGa and PGp) are still to make their way into the relevant TMS literature. However, a number of studies across domains report the Talairach/MNI coordinates of their targeted site, with the exception of the body awareness domain. When including all of the studies, without clustering them into domains, for which standard coordinates of the targeted site were made available (i.e. 23 out of 35) and transforming any Talairach coordinates into MNI coordinates, the mean MNI coordinates (and standard deviations between round brackets) are [44 (4) 65 (7) 36 (8)]. In Fig. 3, the mean coordinates per stimulation site by thematic domain are shown superimposed on a brain template; they are also plotted, along with their standard deviations, on a Cartesian plane. On visual inspection, the mean target sites of language and memory studies, and the mean target sites of visuospatial attention and number processing studies look particularly close to one another. The latter pair is positioned more dorsally than the former pair and shows a large volume of intersection, when considering variability within domains (see Supplementary Fig. 1). The site targeted in motor planning studies appears posterior to both pairs. When examining the mean coordinates separately, along with their variability measures, no clear-cut difference is apparent between domains (see the plot in Fig. 3 and Supplementary Table 2).

### Stimulation protocols and parameters

Online TMS was more frequently used than off-line TMS to manipulate left AG activity, and almost invariably TMS was applied according to a virtual lesion model, with an intent to disrupt its functioning and probe its causal contribution to cognitive functioning, as measured with behavioural indices and self-reports. Of the 35 studies (1 of which had two different protocols and investigated 2 different functions), 25 used repetitive stimulation (8 used 1 Hz, 4 used 5 or 7 Hz, 11 used 10 Hz, 1 used 20 Hz triple-pulse and 1 13.3-Hz triple-pulse, delivering a pulse every 75 ms), 9 adopted cTBS, and 2 spTMS (see Supplementary Table 1 for study methodologies). In three of the thematic areas (memory, motor planning and body knowledge) off-line protocols were dominant, with an overall equal distribution of 1-Hz and cTBS protocols. In the other three thematic areas (language, visuospatial attention, and number processing) online protocols were dominant, with a range of frequencies, 10 Hz being the most popular in the experimental literature and 5 Hz in mapping studies.

### Internal validity controls (site and task)

The majority of included studies had both active control/comparison sites (or a vertex stimulation condition) and control tasks and/or control conditions within tasks, a few studies had only control/comparison sites (or a vertex stimulation condition) and no studies were found having only control tasks. Vertex was most frequently chosen in the memory studies as the single control site in an experimental design.

### Quality

In most cases, details were judged sufficient to reproduce the protocol; however, none of the studies appears to have shared raw data and materials on a public repository. Localization and stimulation methods were also clearly defined. A few studies missed out key sample characteristics, such as handedness and gender composition, and several studies reported information for the originally recruited group of participants but not for the group that was actually included in the analyses. Confidence in AG targeting was variable but overall acceptable according to our established threshold, due to the exclusion of studies unable to identify or check the stimulation site on individual MRI for each participant and/or describing the target region in broad terms. Finally, the robustness of inferential statistical analyses, where performed, was very variable. For example, very few studies performed an a priori power analysis and several studies omitted corrections for multiple comparisons.

## Discussion

In this section, we will review and discuss the evidence found in the 35 selected studies for a causal involvement of the left AG across six thematic areas.

### Language

Eight of the selected studies investigated language-related tasks and can be divided into two main groups: mapping studies and experimental studies (see Supplementary Table 1, Language worksheet).

Mapping studies are of an exploratory nature and geared towards future clinical applications (i.e. they seek to calibrate stimulation parameters for function mapping before neurosurgical excisions). The left AG is but one of their potential sites of interest and this is reflected in the focus of the rationale and reported analyses. Hence, we will limit our inclusion of mapping studies to those that were returned by our initial search (i.e. Hauck et al. 2015; Sollmann et al. 2017; Lin et al. 2017). Here, anatomical localization is based on the cortical parcelation system (CPS) visual template (Corina et al. 2005; 2010). The CPS divides the lateral cortical surface in 37 regions using macro-anatomical landmarks and labels them according to the NeuroNames hierarchy in the Foundational Models of Anatomy NeuroNames (Bowden and Martin 1995; Martin et al. 2001). The regions are identified on each individual brain based on 3D renderings of individual MRI images and localization can be performed a priori or post hoc; when post hoc, it is performed blindly with respect to the errors produced by each stimulation site. Each identified region may contain more than one stimulation site, depending on the density of the grid applied for stimulation. In Hauck et al. (2015), the left AG has the highest number of stimulation sites (five) compared to the other perisylvian regions. In Sollmann et al. (2017) only one site was probed for each selected region (12, including the left AG), whereas in Lin et al. (2017) individual stimulation sites were separated by 0.5 cm and the left AG likely contained several stimulation sites. All three studies used a visual object (picture) naming task. In Lin et al. (2017), participants were asked to always start responding with “This is a…”, to enable a distinction between speech arrest and anomia. Hauck et al.’s (2015) participants also performed the following tasks: pseudoword reading, verb generation (given an object picture), action naming (given a picture of a daily activity, e.g. walking, sleeping…), to assess the validity of the mapping protocol in relation to different language sub-functions. In Hauck et al. (2015) stimulation was delivered at 5 Hz and 7 Hz (100% resting motor threshold, RMT) in separate sessions, starting with stimulus onset and for 1800 ms or about 1300 ms respectively. In Sollmann et al. (2017) it was delivered at 5 Hz (120% RMT) for 800 ms at six different onset times (0, 100, 200, 300, 400, 500 ms after stimulus onset). In Lin et al. (2017) the frequency was individually adjusted to 5 Hz or 7 Hz (the intensity varied between 90 and 130% RMT and was individually adjusted) and lasted for 1800 ms or about 1300 ms from stimulus onset, respectively. Individual adjustment was based on initial checks on the ventral precentral gyrus or the frontal operculum, to optimize stimulation efficacy. Results are typically presented as probability maps, showing error rates for each stimulation site within each region, whereas statistical analyses, when performed, are typically performed without applying corrections for multiple comparisons or collapsed across stimulation sites (and are thus of less interest here). Inspection of the probability map of error rates reported in Hauck et al. (2015) suggests that errors of any category are most frequently elicited by left AG stimulation in the object naming task (15–20% of the trials), and less frequently in the verb generation task (10–15% of the trials) when stimulation is delivered at 7 Hz (but note that a main conclusion of the study is that 5-Hz stimulation is more effective overall). For the left AG, Sollmann et al. (2017) report a preponderance of hesitation errors in object naming (i.e. instances with a delay of at least 200 ms, compared to previous and/or following objects, in the onset of the naming response), except for when the onset of stimulation is at 100 ms after stimulus onset. In that case, no response errors (i.e. a complete lack of naming response) are more frequent, which are considered as analogous to anomia in stroke patients. Finally, Lin et al. (2017) report an unbalanced distribution pattern of essential language sites between frontal (57%), temporal (26%) and parietal (17%) lobes. A role for the IPL is confirmed in some of their participants, with essential sites distributed mainly in the SMG (anterior SMG: 21%; posterior SMG: 29%), whereas only in 14% of the participants the left AG could be classified as an essential language site. Overall, these mapping studies, reporting from an overall sample of 29 German (Hauck et al. 2015; and Sollmann et al. 2017) and 28 Chinese (Lin et al. 2017) participants, suggest that the left AG may be causally implicated in the language function, as probed with an object naming task, in a lower proportion of cases (when considering trials or individuals) compared to neighbouring regions, such as the SMG.

A nuanced picture emerges from experimental studies where either 10-Hz online TMS, where the train of pulses is delivered at or soon after (100 ms) target onset, and ends by 400 ms from target onset, or off-line continuous TBS (cTBS, consisting of 50-Hz bursts of three pulses delivered at 5 Hz for a total of 600 stimuli over 40 s; Huang et al. 2005) have been used to interfere with left AG activity. Three of these studies involve a simple semantic decision on single words (i.e. categorizing auditorily or visually presented words as representing a natural or a man-made object) contrasted with a phonological decision (categorising words as having two or three syllables; Hartwigsen et al. 2010, 2016, 2017) and in one case also with an auditorily or visual perceptual decision task (categorizing words based on the presence or absence of an increase in pitch or in font size towards the end of the word; Hartwigsen et al. 2010). One other study (Sliwinska et al. 2015) requires a simple semantic decision on a pair of visually presented words (i.e. judging whether they mean the same thing or not) contrasted with a phonological decision (i.e. judging whether they sound the same or not) and a perceptual decision on consonant strings (i.e. whether they are identical or not). In all these cases, participants respond by pressing one of two possible response keys with their non-dominant hand (Hartwigsen et al. 2010, 2016, 2017) or with both hands (Sliwinska et al. 2015), and the left SMG is also included in the design as a target region for active TMS. Additional comparison conditions are no TMS trials over the left AG in Sliwinska et al. (2015), bilateral AG and right AG stimulation in Hartwigsen et al. (2010) and sham stimulation in Hartwigsen et al., (2017). In Hartwigsen et al. (2016), the effects of cTBS over the left AG are measured in terms of changes to 10-Hz TMS effects over the anterior inferior frontal gyrus (aIFG) and contrasted with the effects of cTBS over the left SMG as measured in terms of changes to 10-Hz TMS effects over the aIFG. Whereas Hartwigsen et al. (2010) does not report any significant effects of online TMS delivered to the left AG on reaction times (RTs) or error rates in phonological, semantic or perceptual decision tasks, Sliwinska et al. (2015) report slower RTs for TMS compared to no TMS trials on the left AG in the semantic task (synonym judgement) and slower RTs for TMS compared to no TMS trials on the left SMG in the phonological task (homophone judgement). No effects in error rates were detected. For the perceptual task, no effects were detected in both RTs and error rates. It should be mentioned that Sliwinska et al. (2015) employed a TMS-hunting procedure with a visual semantic category judgement task before their experiment, where pairs of words were visually presented, and participants were required to decide whether they came from the same semantic category or not. This procedure enabled Sliwinska et al. (2015) to a) identify in advance, at the individual level, the optimal AG testing site (by probing dorsal, middle and ventral AG sites in each participant, on the basis of coordinates provided in Seghier et al. 2010) and b) discard two participants for whom functional localization failed to identify appropriate testing sites within the AG (two other participants were discarded for failure at the SMG functional localizer, requiring rhyme judgements on pairs of words). Finally, the reported effects (identified in paired comparisons between TMS and no TMS trials per task and site) may not pass the significance threshold after correction for repeated testing (one omnibus ANOVA followed by three split ANOVAs) and multiple comparisons. On the other hand, Hartwigsen et al. (2016) replicate the null result of left AG stimulation (this time applied in the form of cTBS) in a semantic decision task they had reported in Hartwigsen et al., (2010) and concomitantly report a null effect of left aIFG online (10 Hz) TMS on the same task. However, they also document a left AG causal involvement in language-based semantic decisions via its conditioning effects on aIFG sensitivity to online TMS during the semantic task. Indeed, they report slower RTs in the semantic decision task compared to the phonological task when left AG cTBS preceded online aIFG TMS, a difference that was not significant when aIFG TMS was preceded by left SMG cTBS. The authors interpret these results as evidence that both the left AG and the left aIFG are causally involved in language-based semantic decisions but that the functional significance of each region depends on the functional integrity of the other (in other words, the left AG, if unaffected by TMS, could maintain task-function in the presence of a temporary dysfunction of aIFG). In a following study employing the same two semantic and phonological tasks, Hartwigsen et al. (2017) do not find significant behavioural effects of left AG cTBS, compared to sham cTBS or SMG cTBS, on semantic decision RTs, although they document with fMRI significant neural effects of left AG cTBS in both the semantic decision network including the left aIFG (downregulation) and in the phonological decision network including the left SMG (upregulation).

Hartwigsen et al. (2015) stands apart from the previous studies because it employs a more complex task requiring listening to noise vocoded sentences, followed by a vocal response (sentence repetition). The study builds on previous evidence suggesting a crucial role for the left AG in the successful semantic processing of degraded perceptual stimuli, especially when high-level semantic, contextual or combinatorial knowledge would be of help, as is the case with intermediate levels of degradation (Obleser et al. 2007; Binder et al. 2009). In the reported experiment, participants listened to and then repeated a series of noise vocoded sentences of four to nine words each and lasting approximately between 1.5 and 2.8 s. The last word of each sentence was considered a keyword, with low or high levels of predictability based on the preceding context (high: “the storm broke the sailboat’s mast”; low: “the old man thinks about the mast”), and 10-Hz TMS was delivered for 400 ms at keyword onset. Left AG stimulation was compared with a left SPL active control condition. Only intermediate levels of degradation were analysed and a lower, almost null, predictability gain was found at the four-band noise level with left AG TMS when considering the ratio of correctly repeated words per sentence (i.e. considering both the keyword and the preceding words). This effect goes in the expected direction but is significant only before correction. On the other hand, a significantly higher predictability gain with left AG TMS compared to SPL TMS (where it was almost absent) was found at the 8-bands noise level (i.e. with an increased quality of speech signal) when considering the number of correctly repeated keywords. The latter effect was attributed to a decrease, with AG TMS, in the number of correctly repeated keywords for sentences with low-predictable endings, in the condition where a smaller overall predictability effect was present compared to the four-band (more degraded) condition. According to the authors, this pattern of results indicates that, with increasing quality of speech signal, the left AG may become more engaged in a general aspect of semantic processing (i.e. the identification of the correct ending of a sentence). The specific role played by the left AG in language-mediated semantic processing may thus depend on task difficulty.

### Memory

There may be obvious overlaps between language and memory studies and Davey et al.’s (2015) work is one such example (see Supplementary Table 1, Memory worksheet). However, unlike the other studies mentioned in the previous section that use simple semantic tasks, it does not include a phonological task for comparison and it focuses on the organization of stored semantic knowledge. Participants in the study performed two types of word–picture matching tasks (and a matched visual control task) after off-line 1-Hz TMS over the left AG or one of three other sites (vertex, posterior middle temporal gyrus, anterior temporal lobe): one requiring identity matching (where the word could correspond to the exemplar shown in the picture or to a superordinate category, e.g. Corgi or animal) and one requiring thematic matching (where the match is based on either strong or weak association, e.g. Alsatian–bone; Alsatian–razor wire). According to an initial omnibus analysis corresponding to the original design, left AG stimulation appears to more robustly affect the weak rather than the strong association condition and the specific identity condition. However, in follow-up analyses, after recoding the binary association strength variable into a continuous variable and controlling both for non-specific task effects and for effects of stimulus-related variables, the authors find a specific contribution of the left AG to the automatic retrieval of semantic information. This is inferred by significantly slower RTs for stronger thematic associations only. Moreover, a specific contribution of the left posterior middle temporal gyrus to controlled retrieval is also found. This is inferred by significantly slower RTs for weaker thematic associations only. According to the authors, the results emerging from the secondary in-depth analyses is consistent with the known connectivity patterns of the left AG and of the left posterior middle temporal gyrus (default mode network and frontoparietal control system respectively; Davey et al. 2015). Finally, a significant effect of left AG stimulation on the identity matching task for specific, but not for superordinate names is confirmed by the secondary analyses.

Compatible findings were reported by Branzi et al. (2021), who applied online 10-Hz stimulation to the left AG for 400 ms, during a narrative (silent) reading and semantic-integration task of two paragraphs, one providing a context and the other providing target information. Semantically, the initial contextual information could be either strongly or weakly related to the following target information. Stimulation was delivered just before presentation of the target paragraph, that is when contextual–semantic integration was about to take place. Whereas online TMS did not alter reading times during the study phase, it affected a subsequent memory task. More specifically, compared to a vertex stimulation condition, left AG TMS slowed down context-related information recognition for target paragraphs that had been preceded by a highly related context but not for target paragraphs that had been preceded by a weakly related context. Branzi et al. (2021; see also Humphreys et al. 2021), find a similarity between their results and those reported in a range of episodic memory studies (see below) in that they suggest a role for the left AG as a buffering system enabling the online manipulation, retrieval and integration of information that may be stored elsewhere.

Similar to Branzi et al. (2021), Koen et al. (2018) delivered online 10-Hz stimulation to the left AG during the encoding phase. In their case, however, participants had to learn semantically unrelated concrete word pairs. Five-hundred milliseconds after the onset of a word pair, the TMS train was delivered for 400 ms to the left AG or to the vertex. During the study (encoding) phase, participants had to indicate in each trial which of the objects denoted by the words would probably fit into the other. In the following test phase, participants were shown three different types of word pairs and asked to make a memory judgement by judging them as intact (the pair was present in the study phase and also shown in the same pair), rearranged (at least one word was present, but with a different word, in the study phase) and new pairs (the words were not present in the study phase or just one of them was present). The judgement was then followed by a confidence rating (high, low, guess). No effect of the left AG TMS during the encoding phase was found, compared to vertex stimulation, on associative memory performance, which Branzi et al. (2021) related to the lack of a requirement for multi-item, time-extended context integration (for which the left AG may be necessary) in word-pair tasks. However, left AG TMS did affect subjective confidence ratings for incorrectly judged test items, by lowering ratings for associative misses (intact pairs judged as rearranged) and increasing ratings for associative false alarms (rearranged pairs judged as intact). Although the specific pattern of changes is hard to explain, the presence of a significant effect in subjective ratings appears consistent with the findings of off-line TMS studies delivered on the left AG just before a test phase (e.g. Yazar et al. 2014).

The rest of the studies adopted an off-line stimulation protocol of the left AG, using 1-Hz TMS in two cases (Thakral et al. 2017; Wais et al. 2018) and 50-Hz cTBS in five cases (Yazar et al. 2014; 2017; Bonnici et al. 2018; Thakral et al. 2020; Hermiller et al. 2019). In most cases, stimulation was delivered between a learning or study and a subsequent test phase. However, Thakral et al. (2017; 2020) and, in part, Bonnici et al. (2018) did not have a learning or study phase. In such cases, stimulation preceded a cued episodic memory/episodic simulation task or an autobiographical memory task.

Yazar et al. (2014) and Bonnici et al. (2018) employed a similar word-pair association task, though words were presented in the study phase only auditorily by Yazar et al. (2014) and in two modalities (visual and auditory) at the same time by Bonnici et al. (2018). In the study phase, after each pair, participants were asked to form a brief sentence which included both the words and the speaker of the words (previously identified as George, in the case of a male voice, or Olivia, in the case of a female voice). After the study phase, participants received cTBS. After cTBS, they were required to complete a free recall task and a cued recall task. In Yazar et al. (2014), participants were also required to recollect the source (i.e. female or male voice) and to rate their confidence with every judgement. Neither Bonnici et al. (2018) nor Yazar et al. (2014) found any significant effects of left AG cTBS, compared to vertex cTBS, on free or cued recall. Further, Yazar et al. (2014) did not find any effects on source recollection performance but found a significant decrease in source confidence ratings after left AG cTBS compared to vertex cTBS. On the other hand, Bonnici et al. (2018)’s study also included an autobiographical memory task, which was always administered between cTBS and the word-pair test phase (i.e. it was always closer in time to the stimulation). It consisted of a free recall and a cued recall phase on a list of autobiographical events, which had been suggested by the participant at the beginning of the session. Results showed that fewer internal details (i.e. specific details about the events) were reported after left AG than vertex cTBS, whereas stimulation did not affect reporting of external details (i.e. details with no relevance to the event being remembered). Moreover, considerably fewer autobiographical episodes were reported from a first-person perspective after left AG than vertex cTBS. The authors suggest that the evidence is consistent with a specific left AG contribution to episodic recollection in the integration of memory features within an egocentric framework by generating a first-person perspective representation linked to the subjective experience of remembering personal events from the past.

Wais et al. (2018) did not use pairs of words for their study phase but single object pictures. Participants were not informed in advance of the following memory test and during the study phase, while observing each picture, they were asked to respond to questions designed to stimulate an in-depth visualization of the object, such as “yes or no, will the object fit inside a ladies’ medium shoe box?” and, in a separate block, “yes or no, can you carry the object across the room using only your right hand?”. After cTBS on the left AG, on the left IFG or on S1, the test phase started and three categories of object images (targets, lures and novel items) were randomly displayed to participants, who were cued to enter an old/new recognition rating. No significant effect was found that could be related to perturbation of left AG activity in either recognition performance or confidence ratings measures.

Another experiment using object pictures is described in Yazar et al. (2017), where the target object is shown embedded in a natural scene picture and with its name auditorily presented by a male or female voice, having an English or a Scottish accent. After cTBS, the test phase required an old/new recognition judgement to a written word in the middle of the screen. If the word was recognized as old, then a source recollection task followed in three possible versions (administered in different blocks): single source (e.g. gender or side), within-modality double source (e.g. accent and gender), or cross-modality double source (e.g. accent and position). All conditions were introduced as control conditions except for the cross-modality double source condition. Indeed, left AG cTBS did not affect RTs or accuracy levels in source recollection in the control conditions, whereas it appeared to affect, compared to vertex cTBS, source recollection accuracy specifically for the cross-modality double source condition. This could not be attributed to relative task difficulty, as the double source conditions appeared equally challenging.

Thakral et al. (2017) tested participants in three different tasks after 1-Hz TMS to the left AG or the vertex: an episodic memory task, an episodic simulation task and a free associate (control) task. All of these were preceded by a cue word, which had to be repeated aloud by the participant, before starting to remember (episodic memory) or imagine (episodic simulation) and describe from a first-person perspective an event within the past or upcoming 5 years that could incorporate the cue, or to freely generate (free associate) as many words that were semantically/thematically associated with the cue as they could. As expected, no significant difference was found between stimulation conditions in the number of associates produced in the control task. On the other hand, TMS to the left AG, compared to TMS to the vertex, significantly reduced the number of internal details in the memory and simulation tasks (as also expected) and additionally increased the number of external details. The former effect was interpreted as showing a selective impairment, due to left AG TMS, in the generation of internal/episodic details. The latter, unexpected effect, was interpreted as either a compensation effect for the TMS-induced deficit in episodic processing or an altered retrieval orientation strategy. Finally, left AG TMS also seemed to induce a subjective perception of greater difficulty in the episodic memory and simulation task compared to vertex TMS, whereas no such difference was found in the free associate task. A similar episodic simulation task was probed in Thakral et al. (2020), in addition to a divergent thinking task, in an fMRI-neuronavigated experiment, where individual stimulation sites were identified within in the left AG via backtracking from a functionally connected (via resting state network) seed region in the hippocampus. Behavioural results show that following cTBS to the individual-specific target region within the left AG both the number of episodic details produced in the simulation task and idea production (in particular, number of possible uses generated for the cued objects, denoting less fluency) in the divergent thinking task were reduced, compared to vertex stimulation. Unlike in Thakral et al., (2017), this time no difference was detected for external details in the simulation task. Finally, a further study using fMRI-neuronavigated stimulation based on resting state connectivity from a hippocampal seed region, with an a priori constraint on the location of individual stimulation sites within the AG/BA39 region, tested different stimulation protocols over the left AG for their behavioural effects in a memory retrieval task. Hermiller et al. (2019) asked participants to memorize a series of single words shown one by one and with different font shades on a grey background. After cTBS over the left AG region, the authors found a significant improvement in a measure (d’) of recognition performance but not in source recollection accuracy, source memory or any confidence ratings, compared to sham stimulation on the same site. Although left AG stimulation showed an effective interaction with memory task performance, the direction of this finding is at odds with the findings of previously described studies. It has been speculated that this may be due to methodological differences in the choice of comparison conditions and of the tasks used in the test phase (some more taxing on the AG, others more reliant on downstream processing in the hippocampus which may be actually enhanced by cTBS; Thakral et al. 2020). This is, however, an open question.

### Visuospatial attention

Six experimental studies investigated visuospatial attention, adopting a range of TMS protocols (see Supplementary Table 1, Visuospatial attention worksheet). Two studies utilized single-pulse TMS (spTMS) at variable times during the task (Chambers et al. 2004; Schiff et al. 2011), offering insight into chronometry of AG involvement in visuospatial tasks (Sandrini et al. 2011). A further three studies adopted rTMS; two studies delivering 10-Hz rTMS for 500 ms during the experiment, and another study delivering trains of 3 pulses of TMS with pulse gaps of 50 ms (20 Hz) while administering spTMS on V1/V2 in the middle of a train, as part of a phosphene threshold measurement procedure (Silvanto et al. 2009). One study adopted a cTBS protocol (50 Hz off-line), which inhibits cortical activity in stimulated areas, having a disruptive effect (Varnava et al. 2013).

Of the six included studies, all also targeted the right AG, which in some cases is the region of main interest for the authors of the studies, given the substantial literature linking the right PPC with visuospatial attention (Heinen et al. 2011; Uddin et al. 2010; see Bartolomeo et al. 2012 for an overview of networks of visuospatial attention). Thus, in many studies the left may be regarded as a control site; however, all had at least one other condition which could be seen as a control (e.g. another active site or sham stimulation); thus, the main theme of the literature was in elucidating the contribution of a specific cortical subregion or understanding hemispheric asymmetry in visuospatial tasks. The tasks adopted in visuospatial studies varied and included visual search, orienting attention tasks, self-reported phosphene perception and localization, tasks involving stimulus–response correspondence effects, and tasks related to visual neglect.

Of the included studies two investigated AG involvement in orienting attention during a cueing task. The Rushworth et al. (2001) study, which investigated orienting attention, involved participants being instructed to fixate a central cross among four boxes. A warning pre-cue, which involved a box surround turning green preceded the target by 250 ms or 350 ms (the target being the centre of a box turning red). The TMS protocol for this study was rTMS (10-Hz, 500 ms trains delivered 20 ms after target presentation at random for 10% of valid trials and 50% invalid trials) with left AG, right AG, left SMG, and right SMG targeted. Thus, this study also offered insight into the functional specificity of subregions of the IPL in a visuospatial task. The orienting task involved participants responding to a target appearing in one of four spatial locations (four boxes). The pre-cue instructed the participants to orient to one of the four locations. 75% of trials were valid (the target appeared in the same place as the pre-cue), 20% invalid (the target appeared on the opposite side of the pre-cue), 5% no target (the participant had to refrain from pressing a button for 2000 ms). Five participants were tested in this experiment, which found slowing of RTs only after right AG rTMS compared to no rTMS trials, with a significant effect in the invalid trials condition, supporting the right AG involvement in re-orienting attention. There were no significant effects with left AG rTMS on performance in the task. A motor task was included in their following experiment, with the rationale that this would allow for greater insight into the functional specificity of regions in the IPL, with expectations of SMG involvement in this task. This task involved 13 subjects (with 7 or 8 tested at each site, p. 660), where participants were required to press the central pair of keys on a four keypad with the middle and index finger (Home Middle or Home Index). Participants were instructed to respond to the upper target position by pressing the middle finger on the top target key, while simultaneously keeping their index finger on its home key. They responded to the lower target position by pressing down the index finger on the bottom key while keeping the middle finger pressed down its home key. The task involved a pre-cue and a target as in the orienting attention task, but in this task, the pre-cue was valid in 80% of trials and invalid in 20% of trials. Left SMG involvement was supported in the motor task, with rTMS having a disruptive effect on invalid trial responses. As is the case with many earlier TMS studies, the sample size is small. Furthermore, the localization procedure leaves this study just within the boundaries of our inclusion criteria, as the 10/20 EEG system P4/P3 areas were targeted along with a hunting procedure for disruption of visual search. The paper states that in all participants, the critical region was within 1 cm of P4 or P3 and in a portion of the participants the targeted area was checked using MRI after the experiment, while for others frameless stereotaxy was used to locate the stimulated region during the experiment. Thus, as visual anatomical confirmation has been reported with MRI we have included this study; however, the same hunting procedure has been used to guide the targeted area in previous studies and it could be argued that this could lead to areas outside the AG being targeted at an individual level (Ashbridge et al. 1997; Walsh et al. 1999).

Chambers et al. (2004) also investigated the involvement of areas of the IPL (left and right AG, left and right SMG) in orienting attention. In this study, three male participants were tested in a series of sessions with spTMS delivered at one of 12 randomly selected intervals following target onset (30–360 ms) at an intensity of 110% of phosphene threshold. This methodology allowed for the temporal involvement of areas of the IPL to be probed. Adopting a spatial cueing task, where participants made a vertical localization judgement on the left or right of display preceded by a spatially non-predictive cue, the main interest was in performance on invalidly cued trials. In line with the body of literature highlighting a dominant role of the right AG in attention processes (Corbetta and Shulman 2002; Mesulam 1981), the results revealed the right AG was necessary for re-orienting attention, with disruptive spTMS influencing performance in invalidly cued trials between 90–120 ms and 210–240 ms after target onset, with no significant effects of the left or right SMG, or the left AG. Thus, this study highlights the hemispheric asymmetry of the AG in orienting attention. Furthermore, it offers insight into the time points at which the right AG is involved in re-orienting attention, specifically evidencing its involvement at early and later time points during disengagement and re-orienting of attention. The findings support the idea of the AG in the right hemisphere being involved in a network crucial for automatic shifts of attention and suggest it may also be required for processing information of greater complexity during re-orienting to the new target. This study also supports the notion of functional heterogeneity of subregions of the IPL, a brain region noted for its involvement in many cognitive functions (Uddin et al. 2010), and offers greater clarity on the specific role of the AG in visuospatial attention.

Taking the Chambers (2004) and Rushworth (2001) studies together, there is clear support for the involvement of the right AG in orienting attention and differentiation in function for the AG and SMG. The use of different TMS protocols in these studies allows for different insight into IPL involvement in a visual spatial task, with the later Chambers study (2004) not only offering support for the role of the right AG, but also offering greater clarity on temporal involvement of this subregion of the IPL. However, there is little evidence for a critical role of the left AG in orienting attention.

One further study utilizing spTMS and targeting different time points (70, 100, 130, 160, 190, 220, and 250 ms after stimulus onset) during the execution of a visuospatial task is that of Schiff et al. (2011). Targeting the left AG, right AG and vertex and including no TMS trials, the study tested 11 participants and investigated conflict in action selection by adopting a Simon task, where participants responded to a stimulus presented to the left of the right of a central fixation point, in this case an N or an H, based on prior instructions. In this task, half of the participants were instructed to press the leftmost key on the keyboard with the index finger of the left hand when the target letter was an “N” and the rightmost key with the right hand when it was an “H”. The other half were instructed in the opposite target/hand combination. The side of the display on which the letter appears is thus irrelevant to success in the task. However, when the target and response sides match (corresponding condition), RTs are typically faster than when the target appears on the opposite side of the required response (non-corresponding condition), thus revealing a correspondence effect (Umiltà and Nicoletti 1992). In this study, a suppression of the typical correspondence effect was found when TMS was applied to the left and right AG compared with the vertex, but only when pulses were delivered at 130 ms (right AG) and 160 ms (left AG) after stimulus onset. Like the Chambers et al. (2004) study, this offers evidence on the hemispheric contributions of the parietal regions in a visuospatial task, specifically a Simon task, elucidating their temporal involvement. It is somewhat in line with the Chambers et al. (2004) study in terms of the temporal role of the right AG; however, in the Schiff et al. (2011) study, although there was a later time point (250 ms) where a numerical reduction was found for the correspondence effect following right AG stimulation, this was not significant when compared with outcomes following vertex stimulation. In the Chambers et al. (2004) study, a significant right AG involvement in orienting in invalid trials was found between 210 and 40 ms after target onset. It also suggests that in a task where conflict of action is tested, the left AG plays a role, whereas the Chambers et al (2004) study found no evidence to support a role of the left AG in re-orienting attention.

Substantial evidence supports the involvement of the right IPL in orienting attention to the contralateral side, a finding often shown in patients with right IPL lesions (Bird et al. 2006; Mort et al. 2003). Investigating the influence of psuedoneglect, that is, when neurologically healthy participants bisect horizontal lines away from the centre, on the asymmetry induced by a virtual lesion in the parietal lobe, one study delivered cTBS (50 Hz, off-line, 40 s) to either left AG or right AG and included a sham (over the left or the right site) as well as a no cTBS condition (4 separate sessions at least 24 h apart) (Varnava et al. 2013). In psuedoneglect the most common error is leftward; however, individual variability has been reported (see Jewell and McCourt 2000). Adopting a line bisection task, requiring participants to draw a vertical line via a mouse, and a landmark task, requiring participants to press a mouse button to indicate where the vertical transect was in relation to the centre of the horizontal line (left, right, or centre), this study sought to clarify whether both tasks evoke similar results following parietal disruption. Furthermore, as individual differences in the asymmetries shown in pseudoneglect have been evidenced previously, the study sought to investigate whether such pre-existing visuospatial asymmetries are associated with the visual neglect produced by disruption of the parietal regions, namely the left AG and right AG. The study tested 24 participants, with 22 included in analyses for both tasks. As such this is one of the largest sample sizes in the AG-targeted TMS studies on visuospatial neglect; however, with the introduction of groupings based on individual differences in biases related to pseudoneglect, the analyses were based on small sample sizes. Using performance under sham control in the comparative analyses, the results offered no support for left AG cTBS significantly modulating performance in either task. There were significant findings in relation to right cTBS, revealing a significant rightward shift in right deviants, but not in left deviants in this condition compared to the sham condition. The results further support the large body of literature highlighting right hemispheric parietal contributions in tasks of visuospatial attention.

A further study investigated performance in a visual search task (sensitivity to the signal and bias considered), targeting the left AG, right AG, and vertex and including no TMS trials (Muggleton et al. 2008). The left AG and vertex could be seen as control sites, in this study, which tested eight participants in feature and conjunction visual search tasks (5 of the same participants in both). Online rTMS (10 Hz, 500 ms been delivered concurrently with visual search array) over the right AG affected performance in the conjunction search task. The study found no significant effects of TMS over the vertex or left AG relative to no TMS. There was also no effect of any TMS condition on feature search. The findings of this study add to the robust findings for hemispheric asymmetry in visuospatial attention, with a critical role of the AG in the right hemisphere, in contrast to the left.

One final study considered perception of phosphenes following AG stimulation (Silvanto et al. 2009). Thus, this study aimed to elucidate whether the AG exerts a top-down influence on activity in the visual cortex. Silvanto et al. (2009) induced activity in the AG by administering 20-Hz trains of three pulses of TMS with pulse gaps of 50 ms (20 Hz) and administering spTMS on V1/V2 in the middle of a train, as part of a phosphene threshold measure procedure. The study investigated both unilateral and bilateral parietal stimulation during a task, which involved participants reporting verbally whether they perceived a phosphene. Participants were also asked to draw the phosphene(s) they perceived with relation to position in the visual field and shape. Testing seven participants, with only six being naïve to its purpose, the study included a control condition where the pulse train was delivered over the vertex and spTMS on V1/V2 administered in the middle of the train. Considering phosphene thresholds as a percentage of maximum stimulator output, the measurements were calculated relative to baseline threshold. Contrary to right AG stimulation, the effect of left AG stimulation on phosphene thresholds, when considering the right V1/V2, did not reach significance (although did numerically reduce phosphene threshold). rTMS over both the left and right AG (separately) decreased phosphene threshold relative to the vertex condition when considering the left V1/V2. Thus, right AG stimulation exerted an influence on the stimulation required to induce a phosphene from both the left and right visual areas when compared with the vertex condition (reduction). Furthermore, bilateral stimulation had no effect on the stimulation required to induce a phosphene when considering the left or right V1/V2 relative to vertex condition. Shape changes of phosphenes were reported with left AG rTMS for left phosphenes (5/7 subjects) and for right phosphenes (2/7 subjects). Bilateral stimulation was also found to change phosphene shape; however, no details are provided on the number of subjects affected, and vertex stimulation had no effect on phosphene shapes reported. This study offers evidence in line with previous studies highlighting a role of the AG, specifically the right AG, in processing information in both visual fields, whereas, the left AG has been shown to be involved in processing information related to contralateral attention (e.g. Mesulam 1981). The left AG may however play a role in the attentional modulation of activity in the ipsilateral visual cortex, with a consequence in subjective perceptual experience, and in the manifestation of stimulus–response correspondence effects.

### Number processing

There were eight studies that met our criteria for inclusion in the systematic review, five adopted online rTMS (5-Hz or 10-Hz) protocols of varying time windows (Göbel et al. 2001; Rusconi et al. 2005; Göbel et al. 2006; Maurer et al. 2016; Montefinese et al. 2017), one adopted a 1-Hz off-line protocol (Cappelletti et al. 2007), one adopted a 1-Hz off-line and online protocol (Fresnoza et al. 2020), and one adopted a triple-pulse protocol where pulses were delivered at 13.3 Hz (Cattaneo et al. 2009). Four studies considered explicit calculation tasks and four studies looked at number magnitude/numerosity processing or line bisection with a number prime (see Supplementary Table 1, Number processing worksheet).

One of the studies adopted a mapping procedure during calculation, where 52 cortical spots were targeted at 100% individual RMT with online rTMS (5 Hz online for 1.8 s) (Maurer et al. 2016). Individual calculation tasks were displayed for 700 ms with a fixed inter-picture interval of 3 s. The time between task display and the rTMS was 0 ms. The Maurer et al. (2016) mapping study was of an exploratory nature, designed to inform future clinical and scientific endeavours and to investigate the efficacy of the use of repetitive navigated TMS for linking structure with function in different categories of calculation tasks. The 52 targeted spots were targeted three times and chosen based on the findings of previous research regarding discomfort and accessibility. The study required 20 participants to complete twenty simple addition, subtraction, division, and multiplication tasks. For instance, “5 + 6”, “6 – 2”, “1 × 4”, “9: 3”, giving answers verbally. This was carried out at baseline and during mapping. Errors included no response, hesitations, and calculation errors. Notable findings for the left AG revealed a high maximum error rate in the multiplication task (30%). In the subtraction task the highest error rate (for all errors of all stimulations) was in the right AG (13%), followed by the left mMFG and the left AG (10%).

The experimental TMS studies in the systematic review, which related to maths and numerical cognition, also probed the role of the AG in calculation tasks. Fresnoza et al (2020), specifically investigated the role of the left AG and of the horizontal segment of the intraparietal sulcus (hIPS) (vertex used as a control site) in simple multiplication (one-digit × one-digit) and subtraction (one-digit numerals subtracted from tens) tasks, by applying rTMS (1 Hz for 15 min) during the calculation task, which took approximately 13 min in 15 participants. Participants gave verbal answers and performance was also captured before stimulation and at zero, thirty and sixty-minutes following stimulation. Immediately after the experimental session, participants were asked to indicate in a questionnaire how they had solved the problems. Post-hoc analyses on the raw RT data, in relation to the left AG results, showed stimulation of the left AG inhibited the online calculation and retrieval of solutions for multiplication problems, and for subtraction, it inhibited the retrieval (but not the online calculation) of solutions to problems. The information on the strategy adopted was captured post hoc and by self-reported questionnaires, which require participants to be self-aware, to remember with accuracy and to be honest in their reporting, which may be seen as a limitation. The results of this study offer further evidence to suggest the left AG has a role in simple calculation tasks.

Montefinese et al. (2017), also looked at calculation, specifically two-digit mental addition and subtraction, investigating performance during rTMS (10 Hz, online for 300 ms, 4 pulses), delivered 100 ms following stimulus onset, over left and right hIPS and ventral IPS (vIPS) in their first experiment, and over the left and right AG and SMG in the following experiment. Both experiments included the vertex as a control site. Therefore, this study allowed for an understanding of the specific functions carried out by posterior parietal regions of the left and right hemisphere, during calculations that may be considered more difficult than those adopted in the Fresnoza (2020) (a subtraction task that involved one-digit subtracted from tens) and Maurer et al. 2016 (one-digit) studies. The double-digit calculations used in this study involved no carrying/borrowing and included no repeated operands, or operations containing “0” and “1” in the operands or results; participants answered vocally. The study findings related to the AG, evidenced greater hemispheric asymmetry for the SMG than the AG. Furthermore, rTMS effects were higher for the AG in addition than subtraction (in contrast to the SMG). However, rTMS effects were generally stronger for the right hemisphere than the left. The authors suggest that the greater role of the AG in addition, is driven by its involvement in automatic operations as addition is considered a more automatic process than subtraction. The results offer evidence for a role of the left AG in more challenging mental calculations.

A further study investigated the SMG or AG of both hemispheres in three separate experiments while participants carried out a number comparison task, involving double-digit numbers with target numbers between 31 and 99 (Göbel et al. 2001). Participants were required to decide whether the target number was less or greater than 65. The stimuli were displayed until the subject made a response with an inter-trial interval of 1 s. A further experiment in this study adopted a reverse number line, with the participant instructed to press the left button with the left index finger for numbers larger than 65 and press the right button with the right index finger for numbers smaller than 65 (reversed response from the main task). rTMS was delivered at 10 Hz, online for 500 ms before stimulus onset on 10 trials in each experimental block (78 trials, 4 blocks) to nine participants (main number comparison task) and seven participants (reverse number line in experiment 3). It was found that participants responded more slowly following left AG stimulation than without TMS. This TMS-induced increase was greater for numbers greater than 65, particularly those closer to the reference number. A trend was found for increases in RT with right AG stimulation. Participants also responded more slowly with left AG stimulation in the reverse number line experiment, when compared with no TMS. The findings support a role of the left AG in number processing and offer some insight into the nature of its role, with slowed times even in the reverse number condition, suggesting the role is in number processing and not motor response. It should also be noted that, although the procedure used for localizing the AG may raise questions as to whether the left AG was targeted, as a TMS-hunting procedure using visual search (as in Ashbridge et al. 1997) was used, the AG sites were confirmed by MRI scan.

The Göbel et al., (2006) study also utilized the hunting procedure, confirming localization with MRI scans and, as such, has been included. This study targeted the left and right AG and the adjacent posterior part of the IPS (pIPL + S) and SMG and the adjacent anterior part of the IPS (aIPL + S), no TMS trials were also undertaken. The task involved double-digit addition, which involved an addition problem being displayed for 300 ms, a 300 ms interstimulus interval, and two possible answers being displayed for 200 ms. rTMS (10-Hz, 500 ms window) was applied on ten trials in each experimental block. The rTMS stimulation began with the onset of the interstimulus interval and lasted until the onset of the inter-trial interval. Seven participants received stimulation over the left target sites and a further seven participants received stimulation over the right target sites. The study compared stimulation trials with no TMS trials and found that when TMS was applied over the left hemisphere RTs increased from 403 to 419 ms (significant effect); however, there were no significant findings for RTs with stimulation over the right hemisphere. In terms of the differences found between stimulated sites of the left hemisphere, there were no significant findings; numerically, there was a stronger effect for the AG site over the SMG site. This study, therefore, offers evidence to suggest a greater role of left hemispheric parietal regions in double-digit addition but does not offer evidence to suggest subregions of the IPL have functional specificity in this task. It should be noted that Montefinese et al.’s (2015) study also found evidence to suggest the areas of the parietal lobe they targeted (vIPS, hIPS, AG, and SMG of both hemispheres) all had some role in double-digit addition and subtraction but to varying degrees.

Cappelletti et al. (2007), carried out three different experiments each with 12 participants, investigating number magnitude and visual numerosity processing. In two of the experiments, the left and right AG were control sites along with sham on each side of the scalp, and the IPS of both hemispheres was also targeted with off-line rTMS (1 Hz) for 10 min. Participants were tested over two days. In experiment 1, participants viewed centrally presented Arabic numbers (two-digit from 31 to 99) and were asked to judge whether each stimulus was larger or smaller than 65. In experiment 2, participants viewed a reference array of 65 dots, they then viewed ten separate target arrays (31–99 dots) and were asked to decide whether each target array was larger or smaller than the reference array. When comparing with sham stimulation the study found significant findings with IPS stimulation, while rTMS to the left or right AG did not reliably influence magnitude or numerosity comparisons across experiments. This contrasts with the Göbel et al. (2001) study, where left AG stimulation slowed number comparison responses following left AG stimulation when compared with no TMS.

In their study on the association between the fingers and calculation based on the cardinal symptoms of Gertsmann syndrome, Rusconi et al. (2005) carried out a task relating to body awareness, a discussion of which can be found in the relevant section of this review, and a numerical task. The numerical task involved a pair of single digits presented for 65 ms, followed by a 25-ms break and then a two-digit number, which was displayed for 1910 ms. Two versions of the task were used. One of these required participants to ignore the double-digit number and decide whether the two single-digit numbers were smaller or larger than 5. The alternative version required participants to state whether the single digits had the same parity (even or odd). In critical trials, the number to be ignored was arithmetically related to the target digits (via associative multiplication network; LeFevre et al. 1988; Rusconi et al. 2004), and in trials matched to the critical trials this was not the case, but the number to be ignored was matched in terms of parity and (on average) in magnitude. rTMS was delivered at 10-Hz, 500 ms train on 20% of trials of each task in each block and two no TMS blocks were also undertaken. The results support a specific role of the left AG in the task requiring number magnitude processing. Also during left AG stimulation, a significant effect of arithmetical relatedness (mean RT difference between trials with unrelated and trials with related primes) was present in the magnitude task. The finding was also significant when compared with the outcomes of right AG stimulation. A similar outcome was also evidenced in Göbel et al. (2001) where left rTMS delivered at 10 Hz for 500 ms was found to slow down responses in a number comparison task.

The final study considered the role of the AG of both hemispheres in shifts in visuospatial attention brought about by the mental number timeline (Cattaneo et al. 2009). Thus, findings from the studies on visuospatial attention may overlap with this study, which also involves numerical cognition. Testing nine participants (1 left-handed) on a line bisection task, with and without numerical priming, the study sought to disentangle the hemispheric contributions in parietal regions important in visual attention and numerical cognition. The stimuli were horizontally transected lines, with participants being asked to decide which side of the horizontal line is longer, by means of a button press. During the priming condition the target line was preceded by a number prime, which was from one of two ranges of numbers: 16–24 (small prime condition) and from 76 to 84 (large prime condition). A control prime condition was also undertaken, which involved the number primes being replaced with six asterisks. The mental number line was found to influence visuospatial attention, with small numbers biasing attention to the left side of space, and large numbers biasing attention to the right side of space. Triple-pulse TMS was administered with a pulse gap of 75 ms, 0, 75, 150 ms between the prime and the target stimulus. In the small number prime condition, TMS applied over the right AG abolished the effect of number priming, whereas application of TMS over the left AG had no significant effect. In contrast, both left and right AG TMS had an effect on the priming by large numbers. These findings are in line with existing evidence highlighting hemispheric asymmetry in visuospatial attention. That is, as large numbers are mentally represented on the right side of the space, this offers supporting evidence for a role of the right AG in contralateral and ipsilateral attentional processes, while left hemispheric parietal regions are thought to have a role in contralateral attentional processes. The variations in tasks involved and, in their difficulty, make comparisons challenging but offer robust evidence for a role in the left AG for numerical processing, magnitude and calculations.

### Motor planning

Existing research offers evidence for the involvement or association of activity of the AG in preparing for visually guided reach (Castiello 2005; Jeannerod et al. 1995; Vesia and Crawford 2012) and grasp (Randerath et al. 2010). However, many areas within the parietal cortex, specifically the IPL, have been investigated for their roles in motor planning and execution, and it has proven challenging to disentangle the roles of specific cortical subregions. It is a further challenge to understand the connectivity of brain areas during relevant tasks, and the practical limitations of brain imaging methods mean it may be unsuitable for investigating activity during many realistic motor tasks. TMS studies carried out with optimum effective procedures allow for further insight into subregion specificity, offering causal evidence of the role of stimulation targeted areas during motor planning and execution.

The TMS protocols used in these studies (see Supplementary Table 1, Motor planning worksheet) included inhibitory cTBS (50 Hz) delivered off-line for a 40-s window (Adam et al. 2016) and off-line 1-Hz monophasic rTMS delivered for 15 min (Reader et al. 2018). One study tested 16 right-handed participants (Adam et al. 2016), while the other tested 12 participants (10 right-handed and 2 left-handed) (Reader et al. 2018).

Extending current knowledge on the vision-for-action system, Adam et al (2016) set out to further elucidate the contribution of regions in the ventral and dorsal processing streams in visually guided reaching by targeting the left AG, left superior parietal occipital cortex (SPOC), lateral occipital cortex (LOC), a sham location, and also including a no stimulation condition. Adopting a guided reaching task including an egocentric display or no-placeholder condition, which involved participants reaching to a green target appearing by itself, and an allocentric display or placeholder condition with target positions indicated by continuously visible empty placeholder boxes and the target signalled by one of the placeholders being coloured green, participants were required to move their finger as quickly as possible to the target while maintaining end point accuracy. The study evidenced inhibitory cTBS, delivered at 80% RMT over the left AG, eliminated the last target advantage in the placeholder condition. The last target advantage is a deviation from Fitts’s Law, relating movement time to the difficulty of movement (Fitts 1954) and has been evidenced in reach to target tasks where movement time to the last target has been shown to be faster than to the second-last target when placeholders are continuously visible when the task involves targets appearing in horizontally aligned locations to the right of the home position (Adam et al. 2006). In line with Fitts’ law, increased movement times would be expected for further away targets and is seen in unstructured, no-placeholder conditions of the reach task. This was revealed by a one-way repeated measures ANOVA run on the movement time difference scores between the last and second-last target by cTBS condition, showing increments in movement time for the last relative to the second-last target for cTBS over LOC (+ 28 ms) and AG (+ 16 ms); These findings challenge models distinguishing vision for perception and vision for action (Milner and Goodale 1993, 1995) by providing evidence for a role of a region in the dorsal stream (AG) in allocentric coding. The study found no evidence that cTBS over the AG or SPOC disrupted performance in the no-placeholder condition, a finding the authors suggest may be driven by eye movement behaviour, with a free-gaze paradigm being adopted, which may result in participants moving their eyes to the target in peripheral vision, thus making it become a central target. Alternatively, it may be driven by the specific methodology adopted, including factors such as visibility of the hand. For instance, a previous stimulation study has evidenced varied effects of parietal stimulation on outcomes in a memory-guided pointing task depending on visibility of the hand throughout the planning and execution stages (Vesia, 2008). Thus, it is expected that many factors may modulate rTMS outcomes over parietal subregions during motor planning and motor execution tasks, as these regions are highly specified in their function.

Further insights into processing of spatial and temporal information for action in areas of the IPL have been offered by Reader et al. (2018), who investigated the role of the left AG, left SMG, and a no rTMS control condition where little or no stimulation entered the scalp, in types of imitations; specifically, the imitation of emblematic meaningful and meaningless gestures. This study, therefore, offers insight into praxis functions. The stimulation site targeted in this study was specified as central AG, covering part of the anterior (PGa) and posterior regions (PGp). To account for actor bias in digit peak velocity, relative peak velocity of finger movements was considered (the imitator mean digit peak velocity relative to the actor peak velocity) and the findings revealed a significant reduction in relative velocity following inhibitory parietal stimulation regardless of action meaning (small effect size for the left AG). The study did not reveal any effects of parietal stimulation on imitation accuracy, which may be an outcome of the choice of stimulation protocol. For instance, a systematic review of randomized control trials of neurological, psychiatric, and healthy volunteers found no statistically significant effects in any cognitive domain or illness category with slow (≤ 1 Hz) rTMS protocols (Lage et al. 2016).

The two studies utilizing different TMS protocols have shown a role of the left AG in specific functions relating to kinematic processing regardless of action meaning and allocentric processing. The studies support the notion that TMS studies can allow for clarity on specific roles within subregions of the parietal cortex, and with elegantly designed studies TMS can inform existing models. However, it should be noted that in both the studies, there were findings that may have been expected but were unsupported (not all relating to the AG); for instance, with no effects of stimulation on imitation accuracy (Reader et al. 2018) and no evidence that cTBS over dorsal regions disrupted performance in the no-placeholder condition (Adam et al. 2016). Given the methodological differences in the studies, in terms of TMS protocols and tasks adopted, the studies cannot be compared. However, it may be that further methodological studies are required to better understand the best protocols to investigate the AGs role in motor processing.

### Body awareness

This area is represented by two studies, both using 1-Hz off-line TMS (see Supplementary Table 1, Body awareness worksheet). The tasks they employed require some processing of bodily information in order to achieve a successful response and are used to detect cardinal impairments in Gerstmann syndrome (or “angular gyrus syndrome”, see *Introduction*). Indeed, they require intact finger gnosis and left–right discrimination abilities. Unlike studies related to visuospatial attention, the left AG is here expected to play a crucial role and the right AG is included as an extra target site. If involved, the right AG is expected to play a qualitatively different role than the left AG. Rusconi et al. (2005) included also two anterior stimulation sites (left and right SMG), as a check for a possible role of regions involved in motor attention (Rushworth et al. 2001). Their finger gnosis task required responding with a key press from the homologous finger to a vibratory stimulation delivered to one of the ten fingers, while keeping the hands prone and resting on a table with a response button under each finger and the eyes closed. This task was referred to as the opposite-finger key-pressing task and likely requires access to a mental representation of finger identity (e.g. Tucha et al. 1997). In the same experiment, participants also performed a same-finger key-pressing task, which required responding with the same finger that was receiving the vibratory stimulation. This was intended to control for peripheral sensorimotor aspects on the one hand and to bypass the necessity of an internal representation of finger identity for successful task performance (see also Tucha et al. 1997). Rusconi et al. (2005) reported that, when applied either over the left or over the right AG, TMS interfered with the opposite-finger key-pressing task but not with the control task. Whereas the effect of right AG TMS was asymmetrical (i.e. it slowed down responses with the left hand over responses with the right hand), the effect of left AG TMS was symmetrical (i.e. it slowed down responses with the left and the right hand equally). No effects were found on either task after left or right SMG TMS.

Another cardinal symptom of Gerstmann syndrome is left–right disorientation, which is the inability to distinguish the left from the right side on one’s own body or the experimenter’s body (Gerstmann 1940). Hirnstein et al. (2011) thus probed whether the left AG is causally involved in discriminating left from right in healthy participants. They presented their participants with a series of human stickman figures, whose left or right hand was highlighted in red, and with a label indicating “left” or “right” presented at the bottom of the figure. Participants had to decide, by pressing one of two vertically aligned response buttons (with the left hand for half the participants, with the right hand for the other half), whether the label was correct with reference to the side of the red hand, or not. The stickman figure could be depicted facing the participant, and in this case the head was represented as an empty circle, or looking away from the participant, and in this case the head was indicated as a filled black circle. The task was performed in three different blocks: one after left AG TMS, one after right AG TMS and one after sham TMS, with the coil placed over a region positioned midway between the AGs. Although left–right discrimination accuracy was numerically decreased in both active TMS blocks, regardless of stickman orientation (i.e. facing the participants or facing away), only the left AG TMS effect was significant when compared to sham stimulation.

In both cases, therefore, TMS over the left AG interfered in a specific way with performance in a task involving some form of body-related knowledge. Whereas finger gnosis, as tested here, builds on a bodily abstract relational representation that requires to be interfaced with tactile and proprioceptive input, left–right discrimination, as tested here, involves representing bodily sidedness and connecting this framework with both visuospatial and language processes.

## Conclusion

Table 1 offers a closing synthesis of the tasks in which a causal involvement of the left AG has been documented by the reviewed TMS studies. This synthesis is not intended to represent an exhaustive list of tasks where left AG involvement has been claimed. Rather, it is based on a core selection of TMS studies controlling for interindividual anatomical and/or functional variability in their site localization approach. In the remainder of this section, we will offer a concise summary and evaluation of the evidence for the causal involvement of the left AG in the various thematic areas, which may be especially useful in pondering on whether a single functional or computational role may be played by the left AG in all of the reported instances. A possible contribution to any reported behavioural effects from the potential targeting of other PPC regions in at least some of the participants and also from sizeable power issues, indeed, would be more difficult to discard in less specifically targeted or localized stimulation studies (Sack et al. 2009; Ahdab et al. 2010; Button et al. 2013; Karabanov et al. 2019).

**Table 1:** The six identified thematic areas are shown, along with a list of tasks where some evidence of a causal direct involvement or by proxy (via functional connection with distant areas) of the left AG has been reported

Thematic area	Tasks
Language	Degraded speech comprehension (*vocoded sentence*) Object naming *(picture)* Synonym judgement *(pair of words)* Semantic decision *(word)*—via aIFG
Memory	Word–picture identity and thematic matching *(picture and 3 words)* Narrative reading and thematic integration *(written paragraphs)* Source recollection *(object in natural scene picture)* Source recollection confidence rating *(pairs of words)* Episodic memory *(cue word)* Episodic simulation *(cue word)* Autobiographical free recall *(verbal prompt)* Associative recognition confidence rating *(word pairs)* Word recognition *(word)* Divergent thinking *(cue word)*
Visuospatial attention	Simon (stimulus–response correspondence) Phosphene perception—via V1/V2
Number processing	Magnitude comparison *(double-digit vs. reference)* Magnitude matching *(single-digit numbers)* Line bisection with numerical primes *(double digit)* Mental arithmetic *(single-digit and double-digit operands)*
Motor planning	Goal-directed reaching *(kinematics)* Imitation *(kinematics, regardless of meaningfulness)*
Body awareness	Finger gnosis *(tactile stimulus on homologous finger, eyes closed)* Left–right discrimination *(human stick figures facing the participant or away)*

### Single of multiple causal roles in higher functions?

Language is the domain with the most available evidence. On the whole, mapping studies show that, although the left AG may be clearly involved in language at the individual level, it is less frequently confirmed as an essential language region than the adjacent SMG. In particular, the evidence points towards a potential causal involvement of the left AG in visual object naming, as temporary interference with left AG functioning may cause an anomia-like behaviour. However, this does not appear consistent across individuals (in fact, it may not hold true for the majority of tested individuals) or trials. In addition to its practical utility, this evidence urges to take into account individual differences when trying to define the role of the left AG in language. It is perhaps not a coincidence that, among the available experimental studies contrasting simple semantic decisions on verbal material (visually or auditorily presented) to phonological and perceptual decisions, only one that used individualized stimulation sites within the left AG, based on a previous functional localizer, claimed significant behavioural effects of left AG TMS. Such positive result requires replication, due to power issues. An alternative line of evidence, however, suggests that the left AG may be causally involved in a language-based semantic network by virtue of its anatomical connections and functional interplay with the left aIFG. When intact, the left aIFG could compensate for loss of function in the left AG—hence the lack of robust behavioural effects in language-based semantic judgements during or after left AG TMS – and vice versa. An analogous explanation (that is, one suggesting the possibility that the left AG contributes in an adaptive way depending on the context) has been offered for the pattern of results—some of which unexpected—produced by left AG stimulation during auditory exposure to more complex linguistic material whose interpretation may benefit from semantic predictability (noise-vocoded sentences). In this case, however, the role played by the left AG is attributed to the perceptual quality of the speech signals, rather than to the state of a specific node in the network. This does not necessarily contradict the previous model, as it is possible that the functional balance within a language-semantic network is altered depending on the challenge posed by the perceptual context. Overall, the published literature appears to signal that the left AG could be causally involved in visual object naming and in the semantic processing of verbal material although, seemingly, as a supporting actor in most individuals and with a flexible role, depending on the current state of a larger functional network.

Another domain with a good amount of evidence is memory. Broadly speaking, all studies claiming any effects of left AG TMS on some objective measure of performance in memory-based tasks (e.g. speed of semantic matching), reporting indices (e.g. number of relevant events or details produced) and/or subjective confidence measures (e.g. confidence ratings about source judgements) have used exclusively written or spoken verbal materials or pictures in association with written or spoken verbal labels. In many cases, these effects emerge from comparisons with the same task performed during or after vertex TMS. Only studies that did not include a learning phase and were focused on long-term semantic memory, autobiographical memory or memory for episodes (lived or imagined in the future from a first-person perspective), with the exception of a single study on episodic memory which required the semantic integration of narratives, reported detrimental effects of left AG stimulation on memory performance. In these studies, memory performance was variably operationalized as speed of semantic or identity matching, as context recognition speed, or as number of (relevant) reported events or details. On the other hand, when the focus was on episodic associative memory, and stimulation was delivered during or after a learning or study phase, effects were typically not found on memory performance (as measured by free and cued recall, recognition, source recollection accuracy or speed), but rather on subjective confidence ratings for memory-based judgements. In one case, stimulation was reported to affect source recollection accuracy but only if it concerned two different source modalities rather than two source attributes within the same modality. In another case, a paradoxical improvement in item recognition performance after stimulation of the left AG was reported; however, this was attributed to distant effects via angular–hippocampal connections rather than to a local stimulation effect. Thus, the available evidence indicates that performance in memory tasks requiring some form of semantic analysis, semantic or cross-modal integration of materials is related to the integrity of the left AG. The left AG also plays a part in reporting autobiographical or past/future episodic details from a first-person perspective, and in subjective confidence ratings pertaining to episodic associative memory. It should, however, be borne in mind that (a) most of the findings are based on comparisons between left AG stimulation and vertex stimulation b) all of the studies reporting any effects of left AG stimulation are language based or have used visual materials in association with verbal labels. Finally, a systematic introduction of control tasks or conditions for working memory load could help advancing the field by pinpointing the specificity of the left AG contribution to memory processes compared to that of neighbouring PPC regions (see e.g. Berryhill 2012).

The studies included in the visuospatial attention domain had a predominant focus on the AG of the right hemisphere, in line with the right-side dominance posited by successful models of attention and supported by neuropsychological evidence (see Shulman et al. 2010; Husain and Nachev 2007); thus, the left was generally included as another control site to elucidate hemispheric lateralization in visuospatial attention tasks. Taken together, the evidence confirms hemispheric lateralization of the right hemisphere in such tasks. In the two included studies specifically focusing on orientation by using cueing tasks, stimulation of the right hemisphere led to a significant impairment on performance in invalid trials while left stimulation had no significant effect on performance. These findings are also in line with those from studies focusing on conjunction search and line bisection, which showed significant effects of stimulation of right AG stimulation but not left AG. However, a role of the left AG in visuospatial attention emerged in a task involving stimulus–response correspondence and conflict resolution, as stimulation over the AG of the left or right hemispheres suppressed the Simon effect, with the left AG having a later role than the right, and in the perception of phosphenes from VI/V2 stimulation, where left AG stimulation affected the excitability of the left visual cortex whereas stimulation of the right AG affected the excitability of both the left and the right visual cortex. This evidence for a role of the left AG (even though later or more limited than that of the right AG) pops up in two tasks with apparently distinct computational requirements. For instance, the Simon effect likely involves automatic facilitation between a visuospatial signal and a lateralized key-pressing response and conflict resolution between alternative responses, whereas phosphene induction requires conscious access to a visuospatial percept and its translation into a verbal self-report. Overall, given the localization criterion for inclusion in this review, the variation in outcomes is unlikely to result from macro-anatomical inconsistencies in stimulation sites across all of these studies. Nonetheless, it must be considered that the included studies have very small sample sizes (3 > 24; however, these 24 were split into smaller groups during analyses) and are, therefore, likely to lack power, and as single-pulse, multi-pulse, rTMS, and cTBS protocols were used, there is large methodological heterogeneity across studies. Future studies may seek to examine individual differences further to aid in current understanding—the results of the Varnava et al. (2013) study suggest that pre-existing visuospatial asymmetry can influence TMS behavioural outcomes and this could extend to further pre-existing visuospatial biases.

The tasks investigated across all studies in the numerical processing domain included addition, subtraction, multiplication, division, number magnitude comparison, visual numerosity comparison, magnitude matching, parity matching and line bisection with number primes. Elucidating the exact role of the left AG in processes related to these tasks is rendered more of a challenge when considering task difficulty, which also differs between studies and is likely to affect the underlying mechanisms required for task success. Nonetheless, in general terms, the evidence from the empirical studies points to a role of the left AG in mental calculation tasks, with evidence supporting involvement in addition (double-digit), one-digit multiplication, and retrieval of subtraction problems. Many of these findings come from solitary studies when considering task type and difficulty. However, the included mapping study also highlights the high error rates for multiplication induced by left AG stimulation, and although cutoffs are not always clear in mapping studies (Jeltema et al. 2021), taken together, the evidence suggests the left AG contributes to solving multiplication problems. Further evidence has offered support for a role of the left AG in magnitude matching and magnitude matching with priming by a double-digit prime, concomitantly suggesting that stimulation does not prevent automatic arithmetic priming. Left AG stimulation was also found to modulate priming by large numbers (right side of the mental number timeline) in a line bisection task, offering evidence for a role in visuospatial attention, specifically contralateral attention. Furthermore, a study requiring participants to carry out a magnitude comparison task with dot arrays found significant effects following stimulation to the left IPS, but not the left or right AG. It should be noted, however, that the same study did not report any significant effects of left or right AG stimulation in magnitude comparison. On the whole, the evidence from the included studies points to a causal involvement of the left AG in numerical processing. All studies reporting a significant involvement of the left AG presented numbers in Arabic or verbal format. Considering hemispheric lateralization, it appears that the left AG has shown more robust effects across number processing tasks than the right AG. However, the included studies do offer evidence for a role of the right AG in number processing, specifically subtraction (mapping study) and addition; yet, the latter evidence comes from a study which suggests the AG contributes to spatial mapping required by mental addition, more so than mental subtraction. The right AG was also found to contribute in line bisection with priming by small and large numbers, whereas in the same study the left AG was only found to contribute to the large number priming condition, again, relating the right AG to tasks which are likely to involve visuospatial attention. Many of these studies looked to elucidate the contribution of specific parietal subregions, including the SMG and IPS. However, future studies may wish to systematically manipulate factors such as numerical and general task complexity in calculation tasks to help elucidate the specific nature of the role undertaken by the AG of both hemispheres. Finally, investigating different types of non-symbolic and symbolic representations of numerical magnitude might help shed more light on the putative causal role of the left AG in magnitude processing.

The role undertaken by the left AG in motor planning was investigated by two of the selected studies. One of these studies found evidence for a contribution of the left AG in allocentric (i.e. fixed relative to an external landmark) spatial processing, while finding no evidence for a contribution of the left AG in processing egocentric spatial signals (i.e. fixed relative to self) during a reaching task. Considering the possible role attributed to the left AG for encoding events that are relevant from an egocentric perspective within the memory domain these findings are counterintuitive, but may be down to methodological differences in relevant studies; a variation in TMS outcomes has been found depending on whether the target was in foveal vision or extrafoveal vision (SPOC), and whether the hand was visible throughout the task (AG), with visibility of the hand abolishing the effect of TMS, a finding suggested to be driven by the ability to correct errors induced by TMS during task execution (Vesia et al., 2010). Thus, the left AGs role in these processes requires further investigation to elucidate at what stage it makes a contribution and shed light on the role of the dorsal and ventral streams in egocentric and allocentric processing to inform existing models. The evidence considered also supports a role of the left AG in distal movement kinematics, namely reduced peak velocity of the imitator relative to the actor’s during imitation, suggesting it subserves an aspect of motor planning but not imitation accuracy. This is consistent with the outcome of a scoping review investigating noninvasive brain stimulation in limb praxis and apraxia in healthy subjects and patients, finding that, of the different cortical regions targeted, the left IPL was the main area of interest, including the AG and neighbouring regions such as the SMG (Pastore-Wapp et al. 2021).

Finally, two neuropsychologically inspired studies assessed the causal link between left AG functioning and two types of body awareness typically impaired in patients with Gerstmann syndrome. These studies aimed to replicate a behavioural pattern resembling finger agnosia (a pervasive deficit in Gerstmann syndrome, concerning the knowledge of finger identity of either hands) and left–right disorientation (consisting of an inability to accurately identify or recognize body parts according to their sidedness). One study, testing left–right orientation, presented visual stimuli and required language processing, whereas the other, testing finger gnosis, employed tactile stimulation in a non-verbal/non-visual task. In both cases, the evidence appears compatible with the existence of a causal and specific link between the left AG and body awareness. In his early writings, Gerstmann appeared to consider self-evident that left–right disorientation would always be present if a lesion to the left AG caused finger agnosia (Gertsmann 1924, 1927; see also Cubelli and Rusconi 2022). Future TMS studies may help clarify the relation, if any, between different types of body awareness to which the left AG provides a causal contribution.

Taken on the whole, the evidence emerging from this core body of TMS studies indicates that not only the left AG subserves multiple domains, but within domains it may also subserve a range of functions. Although a discussion of current theoretical proposals is beyond the scope of this systematic review, it appears that identifying a single computational process subserved by the left AG (as opposed to just outlining a broad type of functional contribution) that could apply across thematic areas without having to force some of these findings into an uncomfortable box may pose a formidable challenge. The evidence has been considered here for its anatomical relevance rather than for its consistency with a specific theoretical proposal or its collocation within domain boundaries. These core findings appear particularly suited to inform theorization about the possible role(s) of the left AG in higher functions due to the general complementarity of TMS to different methods of investigation, which have produced a large body of evidence, in particular fMRI, and to the site localization approach of the reviewed studies, taking into account at least macro-anatomical interindividual differences.

We will now conclude by highlighting a few limitations and methodological issues, which identify points requiring attention in future theorizing and experimentation.

### Methodological issues, limitations and future directions

In this systematic review, selection criteria were applied to the published literature to capture an inner core of TMS studies that could provide the most specific evidence available on the causal role of the left AG in higher functions in healthy adult participants. An outer core of less specifically targeted or localized, but still potentially informative, literature is not examined here. Most of the retrieved studies present at least a positive finding concerning the effects of TMS over the left AG or a different brain region. Although also a few studies were found in which left AG stimulation led to a null result, it is very likely that this proportion does not fully represent the amount of studies—especially those with the left AG as their main target—finding a null result (see e.g. Chambers 2019). Even if practices are changing in the field and more journals are committed to publish null results, we can assume that the past and current literature still suffers from a large publication bias and null results would be underrepresented in any review of the published literature. On the other hand, we found that some of the positive results published in the literature, on which theories and further experimentation (though never exact replications) may have been based, do not appear to be particularly robust (e.g. main analyses have not been preregistered, positive findings emerge only in post hoc analyses and sometimes only after recoding the original variables, or they would not be deemed as significant if correction for multiple testing was applied, etc.). In general, sample sizes appear to be on the low side, to reliably capture changes in typical cognitive–behavioural effects or measures of performance, whose true effect sizes may be small or medium, and this could raise doubts about replicability of the reported positive findings (Button et al. 2013). Although some of the identified issues are likely related to issues on a larger scale (e.g. the dominant system of incentives in scientific research; Chambers 2019), systematically steering experimentation more and more towards virtuous and ever evolving practices, such as making raw data available in open access repositories and engaging in registered reports and replications (which imply declaring hypotheses before conducting the study, performing a priori power analyses, specifying the primary outcomes and the main analytical strategy beforehand, etc.), could be accessible ways to boost the reproducibility, transparency and self-correction in the field (Hardwicke and Ioannidis 2018; Nature 2020; Munafò et al. 2020; Chambers and Tzavella 2022).

In the reviewed studies, a range of TMS protocols was used. While no TMS protocol is without weaknesses, different protocols are better suited to certain research questions in cognitive neuroscience (see Sandrini et al. 2011, for a discussion). Here, both online and off-line approaches were reported to be effective, and it may not be helpful to suggest certain protocols work better for the investigation of AG functions, as this would depend on the specific question being addressed and on the causal evidence that is already available in the literature within domains. At the level of the thematic domain, there were differences in the most common control and other sites chosen; for instance, in memory studies the vertex was adopted most frequently as a control site, whereas language studies looked at other language areas or utilized sham stimulation over the left AG. Motor planning studies investigated parietal or occipital areas and utilized sham or no TMS conditions, and number processing studies investigated cortical regions within the parietal cortex while investigating hemispheric lateralization by also targeting the right hemisphere. Other control conditions varied and included no TMS conditions, sham and vertex. Body awareness studies utilized a sham, no TMS condition or SMG as a control, while investigating both the left and right hemispheres. Visuospatial attention studies tended to primarily investigate the AG in the right hemisphere, but included the left AG, shedding light on hemispheric lateralization in visuospatial attention. Visuospatial studies often included a no TMS condition, with one also using sham and three using vertex stimulation conditions. Choosing the most appropriate control site is crucial in study design, as targeting an area that is not thought to be involved in a function may still lead to widespread changes within brain networks. For instance, a study comparing supra-threshold TMS (1 Hz for 11 s), administered to the vertex with the same protocol delivered over M1, found no increase in BOLD activation throughout the whole brain; nonetheless, vertex stimulation was shown to induce deactivations in areas of the default mode network, which is important for researchers targeting an area within the inferior parietal lobule (Jung et al. 2016). However, those studies comparing with a sham condition (as obtained for example by placing a tilted stimulating coil over the target area) do not take into consideration the somato-sensory effects of real stimulation, while other sham protocols do; nonetheless, sham protocols still do not allow for a comparison of TMS effects over another active site with the target site (Duecker and Sack 2015). Furthermore, comparison with baseline trials is more extreme in its difference from active stimulation in terms of participant experience, also removing the potential placebo effect that may come with the belief that one is receiving stimulation (Duecker and Sack 2015). These wide variations in methodological decisions pose a challenge for comparing the included studies and may also pose a challenge for future meta-analyses, in addition to the heterogeneity of cognitive tasks and performance measures investigated within domains.

As this systematic review only included studies that utilized localization methods which control a priori for anatomical interindividual variability, we can infer that stimulation was delivered to the targeted anatomical region. However, as pointed out in the results section, there are studies that are on the border of inclusion, namely where a hunting procedure has been used to identify the targeted area, which may, potentially, lead to a hotspot being located outside the target anatomical region. In these cases, studies have only been included where MRI has been used for confirmation of anatomical localization, thus, mitigating the issue of stimulation being administered to a neighbouring region instead of the targeted region. However, as Rugg and King (2018) noted, what we know at the anatomical level strongly suggests that the left AG is a functionally heterogeneous region and may thus subserve a series of functions rather than being devoted to one single domain. TMS studies in different thematic domains may have thus targeted different subregions of the left AG. Here, we did identify, on visual inspection, some topological differentiation between the hotspots associated with research conducted in the different thematic domains, with only number processing and visuospatial attention showing a large volume of intersection (see Supplementary Fig. 1). However, this impression should be taken very cautiously, as target coordinates were available for only a proportion of the reviewed studies and an uneven number of studies contributed to the calculations within each domain. Moreover, when considering variability along each coordinate axis, such differentiation receives no statistical support. Still, even in the presence of a genuine topological differentiation of the causal role of the left AG across thematic domains, we could not discard the possibility that the left AG specializes in a specific type of computation that could be then applied with adaptations across domains. A step forward to solving the issue could come from systematically probing the same AG hotspot for its causal role in different domains within study and within participant. Moreover, instead of (or in addition to) choosing an active control site outside the left AG, a distinct part of the same region could be chosen to act as a control (e.g. PGa for PGp, and vice versa). Also within single domains, it is still debatable whether the left AG subserves a single function or multiple functions, or whether its contribution may flexibly change depending on current computational requirements. It is possible that one key to greater clarity is to take into account that different parts of the same macro-anatomical area may be involved in separate functional networks (Uddin et al. 2010) on the one hand and to refine our analysis at the level of interindividual differences: for example, by systematically factoring in interindividual variability in area boundaries (as suggested by Rugg and King 2018) and/or interindividual functional variability on top of anatomical variability at the design stage. For instance, variations in functional variability may result in high variability in the affected brain networks, and in turn, the behavioural outcomes of TMS protocols (e.g. Harita et al. 2022). This potential issue may be helped in part by using fMRI-guided TMS neuronavigation. Reporting of responsiveness at the level of the participant may also allow for a better understanding of the effects of TMS on targeted sites, thus allowing for a better understanding of their function (see Lowe and Hall 2018 for an overview).

## Supplementary Information

Below is the link to the electronic supplementary material.

Supplementary file1 (DOCX 202 KB)

Supplementary file2 (XLSX 65 KB)

Supplementary file3 (DOCX 138 KB)

## Data Availability

This paper does not have data attached. The resources created for and obtained from this study are available on the Open Science Framework at the following link: https://osf.io/8f2m3/.
