# Supplementary material for: Causal involvement of the left angular gyrus in higher functions as revealed by transcranial magnetic stimulation: a systematic review
Source: Brain Struct Funct. 2022 Oct 19;228(1):169–96. doi: 10.1007/s00429-022-02576-w (PMC9813212; doi:10.1007/s00429-022-02576-w)
Supplement: Supplementary file 1 — Supplementary file1 (DOCX 202 KB) [file 429_2022_2576_MOESM1_ESM.docx]

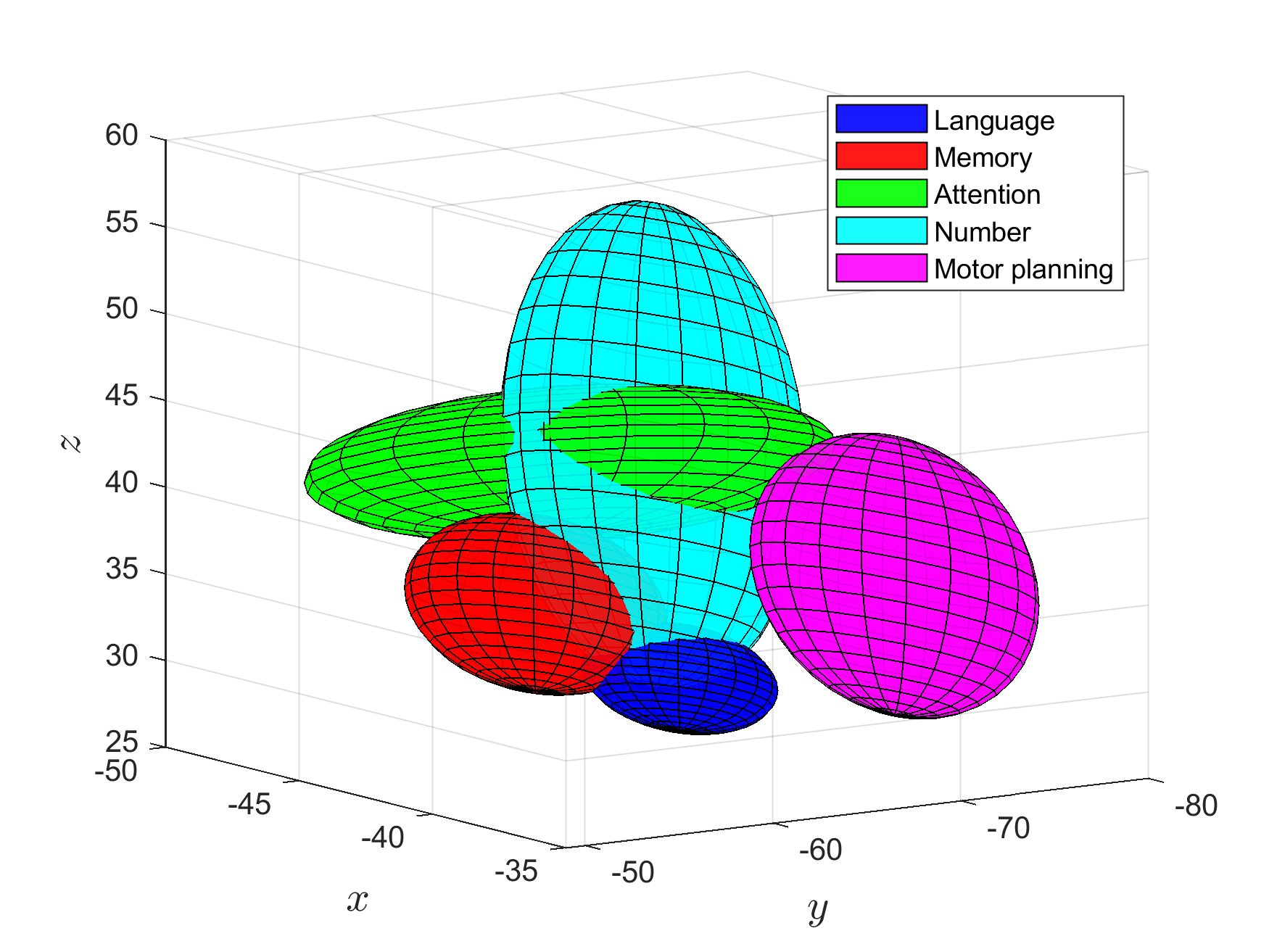


**Supplementary Figure 1.** MNI mean coordinates and their standard deviations have been plotted on a 3D Cartesian system to form volumetric “hotspots”, centred on means and with standard deviations as radii. Domains have been color-coded, to help appreciating the relative position of their respective “hotspots”.
