# Supplementary material for: Causal involvement of the left angular gyrus in higher functions as revealed by transcranial magnetic stimulation: a systematic review
Source: Brain Struct Funct. 2022 Oct 19;228(1):169–96. doi: 10.1007/s00429-022-02576-w (PMC9813212; doi:10.1007/s00429-022-02576-w)

**Supplementary Table 2.** Mean coordinates and standard deviations (the latter between brackets) are shown by thematic domain.


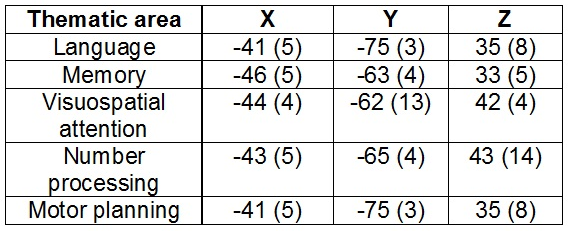

Supplement: Supplementary file 3 — Supplementary file3 (DOCX 138 KB) [file 429_2022_2576_MOESM3_ESM.docx]
